# Do improvements in infant and young child feeding (IYCF) practices endure the test of time?

**DOI:** 10.1186/s41043-024-00507-5

**Published:** 2024-04-05

**Authors:** Solis Winters, Sebastian Martinez, Julia Johannsen

**Affiliations:** 1 School of Public Health, University of California Berkeley, Berkeley, CA USA; 2 International Initiative for Impact Evaluation (3ie), Washington, DC USA; 3 Inter-American Development Bank, La Paz, Bolivia

## Abstract

**Background:**

Reducing malnutrition is a key priority for governments in low- and middle-income countries given its lasting effects on child development, health, income, and economic growth. Strategies to improve recommended infant and young child feeding (IYCF) practices, especially during the first two years of life, are considered among the most effective.

**Methods:**

In this paper, we evaluate the long-run impacts of an innovative education strategy based on interactive play and performing arts implemented in El Alto, Bolivia on caregivers’ IYCF knowledge and practices. Two thousand and fifteen households were randomly assigned to intervention and control groups. Two rounds of data were collected approximately 30 and 42 months after baseline. We estimate short-term (30 month) and longer-term (42 month) intent-to-treat effects using multivariate linear regression analysis, with and without controlling for covariates.

**Results:**

The program significantly increased caregiver IYCF knowledge by 0.13 SDs in the short run, and this effect grew over time. The program also improved adherence to recommended IYCF practices by 0.23 standard deviations (SDs) in the short term, but the effect on practices dissipated over time, and no longer-term impacts were detected. Caregivers with above median baseline knowledge, number of children, and age appear to have benefited most from the program.

**Conclusions:**

Our findings suggest that entertainment-education interventions are a promising model for improving and maintaining IYCF knowledge. However, their ability to sustain more permanent changes in IYCF practices is less certain. Further evidence is needed to identify other avenues for producing long-term, sustainable behavior change, especially among indigenous populations in Latin America, where literature on education and behavior-change interventions related to IYCF practices is limited.

**Supplementary Information:**

The online version contains supplementary material available at 10.1186/s41043-024-00507-5.

## Introduction

Child malnutrition remains a highly prevalent and pressing issue globally, causing 45 percent of all deaths in children under five—about 3.1 million each year [1]. The effects of malnutrition are often irreversible and can persist for generations. Poor nutrition before two years old causes impaired physical and cognitive development, resulting in lower IQ, decreased attention, greater learning difficulty, increased behavioral and social problems, and lower educational achievement [2–7]. Malnutrition also has long-term consequences on health, income, productivity, and economic growth [2, 3, 7]. For these reasons, addressing early child malnutrition is a global development goal and a key priority for governments in low- and middle-income countries.

It is well known that pregnancy and the first two years of life provide the greatest window of opportunity for improving child nutrition and growth [2, 3, 7, 8]. Strategies to improve recommended infant and young child feeding (IYCF) practices are considered among the most effective [7–9]. The benefits and importance of IYCF practices, such as exclusive breastfeeding and timely and adequate introduction of complementary foods, are well documented, but gaps in knowledge about these practices among caregivers continue to be a major barrier to their adoption [7–10].

Education interventions have been successful in increasing knowledge and adoption of recommended IYCF practices. Programs that incorporate flexible communication strategies and are adapted to the context and culture show the greatest success in changing behavior. Additionally, interventions that address both intrapersonal factors (knowledge, attitudes, and beliefs of individuals) as well as interpersonal and environmental factors (norms, beliefs, and policies within families, communities, and institutions) and make use of social networks tend to be more effective than those focused only on the individual. Education programs implemented in developing countries have also found greater effects on adoption of recommended practices compared to those delivered in developed countries [8, 9, 11–17]. The effects of education interventions on child growth and nutrition are mixed. While some studies found significant reduction of stunting and improved height and weight, others found changes only in one measure of growth, or no significant effects [15–20].

In Latin America, improving child nutrition continues to be a major priority. Governments in the region are investing in infant and young child feeding, but evidence for education interventions in Latin America, and particularly among indigenous populations, is limited. Entertainment-education provides a promising opportunity for behavior change within this context. Entertainment-education aims to increase knowledge, change attitudes, and promote social changes through different forms of entertainment. Entertainment-education interventions target behavior change at the group or community level, focusing on social interactions, networks, and norms. Experimental evidence on the effect of entertainment-education on health is limited, but preliminary research shows its potential in changing health-related behaviors and improving health outcomes [21]. Given its roots in oral and performing arts traditions such as storytelling and theater, entertainment-education may also provide a more culturally adapted and effective approach for indigenous populations compared to other, more traditional education strategies [22–24].

In this paper, we evaluate the longer-run impacts of an innovative education strategy based on interactive play and performing arts implemented in El Alto, Bolivia, on IYCF knowledge and practices. We assess heterogeneous program effects by caregiver and household demographics. Our findings add to the literature on entertainment-education interventions, particularly in the context of Latin America and among indigenous populations, where evidence for IYCF education interventions is limited [25–27].

## Methods

### Ethics

The study survey was reviewed and approved by the ethical review board of the National Bioethics Committee of Bolivia at the National University San Simon. Informed consent was obtained from parents and guardians before data collection and access to identifiable data was restricted to maintain confidentiality. De-identified microdata are publicly available at http://dx.doi.org/10.18235/0001649.

### Study design

We conducted a randomized control trial in the 8th district of El Alto, Bolivia, to evaluate the effect of a community nutrition program on caregiver knowledge, IYCF practices, and child nutrition and growth [26]. The city of El Alto, located adjacent to La Paz in the Altiplano highlands, is the fastest growing urban center in Bolivia with the highest population density in the country. In 2016, prior to the intervention, the city had a poverty rate of 67% and was 86% indigenous, the majority identifying as Aymara. The 8th district, where the program was implemented, was predominately low-income. At baseline, most families lived in precarious housing conditions, had no sanitation or waste collection services, and exhibited poor health practices [25].

**Fig. 1:**
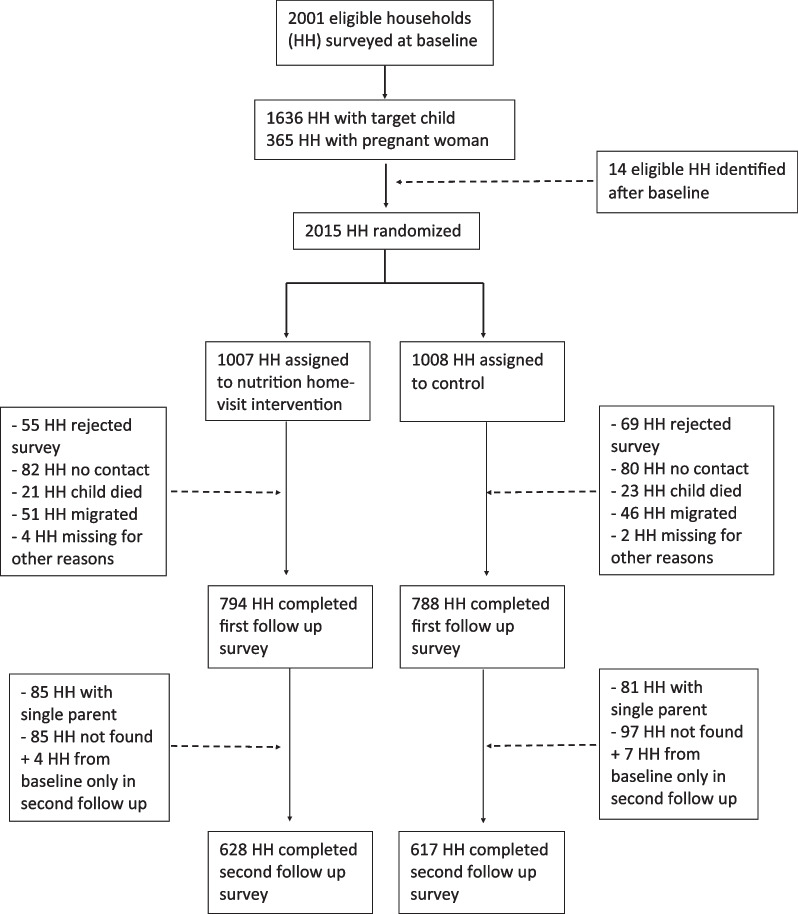
Trial profile

Prior to the start of the program, the research team conducted a census in the 8th district to identify households with a pregnant woman or a child under 12 months old. Two thousand and one households were eligible and baseline data were collected between March and July 2014. Following baseline, the study randomly assigned households to treatment and control groups with equal probability. Fourteen additional households were recruited after baseline and were randomized according to the same procedure. The project invited households assigned to treatment to participate in the program and enrolled a total of 1882 consenting households. Two rounds of follow-up data were collected approximately 30 months and 42 months after baseline. Martinez et al. 2018 [26] presents the results of the 30-month follow-up. This study adds a second round of follow-up data 42 months after baseline and expands the original analysis with a focus on IYCF. The study design is illustrated in Fig. 1.

### Description of Intervention

The Community Child Nutrition (CCN) project was implemented by the Andean Rural Health Council (CSRA, for its Spanish acronym) between 2014 and 2016 [25, 26]. The intervention used a culturally adapted entertainment-education approach to promote recommended nutritional practices and built upon previous experiences of CSRA with nutrition counseling in El Alto [26, 27]. The project’s objectives were to improve IYCF practices, hygiene, and nutritional status through a behavioral-change education strategy based on participatory play. The project hired community health workers to deliver the program. Health workers were required to have at least three years of work experience in family and community health, some work experience with entertainment-education, residence in El Alto, and understanding of the native language (Aymara). Most health workers also had formal training as doctors or nurses. Following recruitment, the program trained health workers on nutrition, according to Ministry of Health standards, and on management, application, and creation of program scripts and play techniques (puppets, clowns, songs, poems, communication skills, construction and acting of characters). Recruitment and training of health workers lasted about three months.

After the training period, health workers delivered the behavioral-change strategy to caregivers and family members in enrolled households. Home visits were organized along age-specific curricula called “educational paths,” which emphasized key nutrition messages caregivers should receive based on the child’s age at each stage of the intervention. During each visit, educational paths emphasized one message from the following areas: exclusive breastfeeding and complementary feeding, responsive feeding, and hygiene (with an emphasis on handwashing). The program staff delivered messages face-to-face with the use of interpersonal communication and participatory demonstrations. They enhanced the intervention by conveying information through interactive play and performing arts (puppet shows, theater, songs, or poems). These communication strategies engaged families and caregivers at a cognitive and emotional level. Home visits lasted between 60 and 90 min and occurred once or twice per month depending on the child’s age. Children graduated from the program at two years old, regardless of their age at start.

### Data collection

Household surveys were collected at baseline, during the first follow-up (about 30 months after baseline), and during the second follow-up (about 42 months after baseline). The research team conducted interviews with the mother or primary caregiver of every target child (child < 12 months old at baseline). They recorded information on household sociodemographic and caregiver characteristics. In both follow-up surveys, caregivers were asked detailed questions about their feeding practices and the target child’s intake of individual food groups within the last 24 h, in accordance with WHO IYCF guidelines [28]. Caregivers were also asked questions to measure their knowledge of IYCF and nutrition practices promoted by the program. The team also collected administrative data including information on program implementation, enrollment, participation, and graduation.

### Sample size and power

Two thousand and one eligible households completed the baseline survey, 1582 households completed the first follow-up survey, and 1245 completed the second follow-up survey. One thousand two hundred and thirty-four households completed both follow-up surveys. Power calculations were performed as part of the study design. Ex ante, the study was powered (power of 0.80 and significance of 0.05) to detect a 5 percentage-point change in stunting or a 0.13 SD change in height-for-age z score [25]. Ex post power calculations on the first follow-up yielded a minimum detectable effect size of 0.14 SDs in height-for-age z scores for the intent-to-treat (ITT) sample [26].

### Outcome definitions

The primary outcomes of interest for this study are defined as follows:

- *Knowledge index* a score between 0 and 1, representing the proportion of correct responses to 43 knowledge-based questions regarding IYCF practices. All 43 components included in the index are the same between rounds. A list of individual components is presented in Additional file 1: (S1 File).
- *Practice index* a score between 0 and 1, representing the probability of following recommended IYCF practices. Components included in this index vary between data collection rounds due to age-specific practices. The individual components included in each round are presented in Additional file 1: (S2 File).

Each index averages the components of equally weighted questions as follows:${index}_{i}=\frac{\sum_{j=1}^{J}{X}_{ij}}{J}$, where ${index}_{i}$ is the index score for household $i$ on question$j$, ${X}_{ij}$ is a binary variable equal to 1 if the caregiver responded according to recommended practices and 0 otherwise, and $J$ is the total number of items in the index. Standardized versions of the indices were constructed as follows: ${{z}_{{\text{index}}}}_{i}=\frac{\left({{\text{index}}}_{i}- {{\text{index}}}_{c}\right)}{{{\text{SD}}}_{c}}$, where ${z\_{\text{index}}}_{i}$ is the z score for ${\text{index}}$ in household$i$, ${{\text{index}}}_{c}$ is the mean of $index$ in the control group, and ${{\text{SD}}}_{c}$ is the standard deviation of ${\text{index}}$ in the control group.

### Statistical analyses

Due to limited program funds, data for the second follow-up were collected by another program that, based on its design, was only interested in households with two parents. This posed an empirical challenge for our analyses since households with both parents may have had underlying differences in demographic, socioeconomic, and health characteristics compared to single-parent households. In order to minimize potential bias due to unobserved differences between households across rounds, we limited all analyses to the balanced panel of two-parent households that were reached for second follow-up (1225). We ran balance tests for this subsample of households to check whether demographic and socioeconomic characteristics at baseline were similar between treatment and control groups. We confirmed that the balance expected from randomization of treatment holds for this subsample. We also ran balance tests between treatment and control groups for households that attrited, by design or otherwise, to check for selection bias due to attrition. Additionally, we replicated all analyses with the full sample of households to see whether our results are robust to different sample specifications. Tables with these results can be found in Additional file 1.

To compare differences in average outcomes across treatment and control groups, we estimated ITT effects based on the initial random assignment using multivariate regression analysis, with and without controlling for covariates. ITT effects were estimated using the following model:

$${Y}_{i}= \alpha + \beta {T}_{i}+ \sum_{j=1}^{n}{X}_{ij}+ {\epsilon }_{i}$$

where ${Y}_{i}$ is the outcome of interest for household $i$, ${T}_{i}$ is a binary variable equal to 1 if assigned to the treatment group and 0 if assigned to the control group, $\beta$ is the average ITT effect, and ${X}_{ij}$ is a vector of $j$ covariates included in the regressions.

We also ran the same regression models with inverse probability weights to account for potential selection bias due to sample attrition. Weights are calculated as follows: ${w}_{i}=\frac{1}{\widehat{p}({x}_{i})}$, where ${w}_{i}$ is the imputed weight for household $i$, ${x}_{i}$ is a binary variable equal to 1 if household $i$ was observed in all rounds (not attrited) and 0 otherwise (attrited), and $\widehat{p}({x}_{i})$ is the estimated probability of being observed after controlling for household and caregiver characteristics.

Additional regressions were performed with interaction terms to assess potential heterogeneous ITT effects. Caregiver age (years), number of children, baseline knowledge (probability), and education (years) were dichotomized at the median and used in the interactions. The baseline knowledge indicators include 106 observations for children who were not the target child (older siblings). A dummy variable controlling for the change in the target child was added to the adjusted regression models for these indices. All statistical analyses were conducted in Stata (V.15.0).

## Results

### Study population

Table 1 describes the baseline descriptive statistics for caregivers, spouses, children, and households for the balanced panel of two-parent households. In households where the child was not yet born, the pregnant mother was assumed to be the child’s caregiver. Age at baseline (negative), gender, and indigenous status for the children in utero were taken from the first follow-up data and included in baseline descriptive statistics.

**Table 1:** Baseline characteristics

	Treatment			Control			Balance		
	n	Mean/Proportion	SD	n	Mean/proportion	SD	Effect	Lower bound	Upper bound
*Caregiver characteristics*									
Female = 1	618	0.998	(0.04)	607	0.998	(0.04)	0.000	− 0.005	0.005
Age in years	618	27.898	(6.64)	607	27.812	(6.46)	0.086	− 0.648	0.820
Indigenous = 1	618	0.845	(0.36)	607	0.825	(0.38)	0.019	− 0.022	0.061
Education in years	618	8.816	(3.96)	607	9.227	(3.72)	− 0.412*	− 0.843	0.019
Can read = 1	618	0.984	(0.13)	607	0.992	(0.09)	− 0.008	− 0.020	0.004
Worked in the past week = 1	618	0.296	(0.46)	607	0.280	(0.45)	0.016	− 0.035	0.067
Married or with partner = 1	618	0.963	(0.19)	607	0.964	(0.19)	− 0.001	− 0.022	0.020
Biological parent of target child = 1	618	0.922	(0.27)	607	0.934	(0.25)	− 0.012	− 0.041	0.017
Household head or spouse of head = 1	618	0.934	(0.25)	607	0.929	(0.26)	0.004	− 0.024	0.033
*Spouse of caregiver characteristics*									
Female = 1	597	0.002	(0.04)	585	0.002	(0.04)	− 0.000	− 0.005	0.005
Age in years	597	31.003	(7.89)	585	30.773	(7.41)	0.231	− 0.643	1.105
Indigenous = 1	597	0.843	(0.36)	585	0.826	(0.38)	0.017	− 0.026	0.059
Can read = 1	596	0.997	(0.06)	584	0.998	(0.04)	− 0.002	− 0.007	0.004
Education in years	597	10.446	(3.32)	585	10.660	(3.32)	− 0.214	− 0.593	0.165
Worked in the past week = 1	597	0.958	(0.20)	585	0.957	(0.20)	0.001	− 0.022	0.024
*Target child characteristics*									
Female = 1	618	0.484	(0.50)	607	0.519	(0.50)	− 0.035	− 0.091	0.021
Age in months	618	4.704	(5.02)	607	4.517	(4.98)	0.187	− 0.374	0.747
Indigenous = 1	618	0.595	(0.49)	607	0.573	(0.50)	0.022	− 0.033	0.077
*Household characteristics*									
Household size	618	4.822	(1.69)	607	4.789	(1.73)	0.033	− 0.159	0.225
Number of rooms	618	1.859	(1.01)	607	1.932	(1.14)	− 0.073	− 0.194	0.047
Monthly per-capita income (Bs)	618	625.687	(558.79)	607	591.823	(493.88)	33.864	− 25.224	92.953
Water = 1	618	0.243	(0.43)	607	0.257	(0.44)	− 0.014	− 0.063	0.034
Electricity = 1	618	0.997	(0.06)	607	0.997	(0.06)	0.000	− 0.006	0.006
Home phone = 1	618	0.029	(0.17)	607	0.023	(0.15)	0.006	− 0.012	0.024
Cellphone = 1	618	0.977	(0.15)	607	0.975	(0.16)	0.002	− 0.015	0.019
Kitchen = 1	618	0.997	(0.06)	607	0.992	(0.09)	0.005	− 0.003	0.013
Radio = 1	618	0.848	(0.36)	607	0.832	(0.37)	0.016	− 0.025	0.057
Television = 1	618	0.972	(0.16)	607	0.974	(0.16)	− 0.001	− 0.019	0.017
Refrigerator = 1	618	0.191	(0.39)	607	0.189	(0.39)	0.001	− 0.043	0.046
Vehicle = 1	618	0.254	(0.44)	607	0.269	(0.44)	− 0.014	− 0.064	0.035
Water pump = 1	618	0.002	(0.04)	607	0.003	(0.06)	− 0.002	− 0.007	0.004
Air conditioning = 1	618	0.000	(0.00)	607	0.002	(0.04)	− 0.002	− 0.005	0.002
Computer = 1	618	0.120	(0.32)	607	0.120	(0.33)	− 0.001	− 0.037	0.036
Bathroom or latrine = 1	618	0.872	(0.33)	607	0.852	(0.36)	0.020	− 0.018	0.059
Sewerage connection = 1	618	0.346	(0.48)	607	0.376	(0.48)	− 0.029	− 0.083	0.025

Significant effects are in bold font; ***, **, and * indicate *p* < 0.01, *p* < 0.05, and *p* < 0.1, respectively. Binary variables are presented with “ = 1” and proportions are reported in the table

Most caregivers are female (> 99%) and indigenous (84%). At baseline, caregivers had nine years of education and were 28 years old. About 30% worked at least one hour in the week prior to the survey. Most were married or in a domestic partnership (96%) and identified as the biological parent of the target child (93%). On average, their spouses were slightly older (31), slightly more educated (11 years), and had much higher rates of employment (96%). Children, including those in utero at baseline, were on average 5 months old. About half are female and 58% belong to an indigenous group.

Households had an average of 5 members and 2 rooms. The average monthly per-capita income per household was about 600 Bolivianos ($86USD). Most households had electricity (> 99%), a kitchen (> 99%), a cellphone (98%), a radio (84%), a television (97%), and a bathroom or latrine (86%). Just over a third had sewerage connection and about a quarter had a vehicle and running water. 19% had a refrigerator and 12% had a computer. Very few households had a home phone (3%), a water pump (< 1%), or air conditioning (< 1%).

### Balance

To verify that there were no significant differences between treatment and control groups at baseline, we conduct balance tests for a variety of household demographic and socioeconomic characteristics. The results presented in Table 1 confirm that baseline characteristics were well balanced between treatment and control groups.

### Attrition

Table 2 presents sample attrition among two-parent households, including those who rejected the survey or could not be reached, rather than those who were systematically dropped (single-parent households). Attrition among these households was about 22% between baseline and the first follow-up and about 12% between the first follow-up and the second follow-up. The sample attrition rate was about 31% in total and was balanced between treatment and control groups. The attrition rates for the full sample of households can be found in the (Additional file 1: Table S1), along with tests for balance of demographic characteristics among attrited households (Additional file 1: Table S2).

**Table 2:** Sample attrition among two-parent households

Attrition	Treatment		Control		Effect	Lower bound	Upper bound	*p* value
	Mean	SD	Mean	SD				
Baseline to Follow-Up 1	0.217	(0.41)	0.224	(0.42)	− 0.007	− 0.045	0.031	0.723
Baseline to Follow-Up 2	0.307	(0.46)	0.321	(0.47)	− 0.014	− 0.057	0.029	0.526
Follow-Up 1 to Follow-Up 2	0.120	(0.33)	0.137	(0.34)	− 0.017	− 0.052	0.018	0.336
Total	0.310	(0.46)	0.329	(0.47)	− 0.020	− 0.063	0.023	0.371

Significant effects are in bold font; ***, **, and * indicate *p* < 0.01, *p* < 0.05, and *p* < 0.1, respectively. Attrition rates are computed among two-parent households to capture attrition that was unplanned, rather than households which were dropped by design

### Compliance

We used administrative records and self-reports to determine treatment compliance. Two control households were unintentionally enrolled in the treatment group. At the first follow-up, 66% of all households in the treatment group completed the program (child graduated). Among the 34% that did not complete the program, 22% were enrolled but had not completed the program and 12% rejected enrollment after baseline. In total, 73% of households assigned to treatment and 6% assigned to control self-reported participating in the program. Although the study was randomized at the household level in the same district, only 9.7% of households assigned to control reported hearing about the program.

### Intention-to-treat (ITT) analysis

This section presents short- and long-term ITT effects of the program on caregiver IYCF knowledge and practice, where short term refers to the first follow-up (30 months after baseline) and long term refer to the second follow-up (42 months after baseline). The effect of the program on caregiver knowledge is presented in Table 3. In the short term, the program increased caregiver knowledge by about 0.134 SDs, when controlling for caregiver age and education, household income, household size, and water and sanitation. More specifically, the program improved knowledge surrounding breastfeeding, complementary feeding, and the benefits of micronutrients by 0.129, 0.287, and 0.120 SDs, respectively. No significant effects were found with respect to knowledge of adequate diet during pregnancy or the benefit of eating animal products. Overall knowledge decreased for both groups in the long term, but the knowledge gap between caregivers in the treatment and control groups grew to 0.269 SDs. Knowledge of complementary feeding and the benefits of micronutrients were sustained in the long term, but the effect of the program on breastfeeding knowledge dissipated. Additionally, caregivers who received treatment had greater knowledge of recommended diet during pregnancy and the benefits of animal products in the long term, of 0.129 and 0.132 SDs, respectively. These results are robust to the inclusion of inverse-probability weights and are consistent with the full-sample estimates (Additional file 1: Tables S3 and S4).

**Table 3:** ITT effects on knowledge

	Treatment			Control			Adjusted		
	n	Mean	SD	n	Mean	SD	Effect	Lower bound	Upper bound
*Short term*									
Knowledge index	589	0.486	(0.09)	587	0.473	(0.10)	0.013**	0.002	0.024
Standardized knowledge index	589	0.183	(0.95)	587	0.057	(0.99)	0.134**	0.023	0.244
Diet during pregnancy	589	0.612	(0.19)	587	0.612	(0.20)	0.001	− 0.021	0.024
Diet during pregnancy (standardized)	589	0.036	(0.95)	587	0.034	(0.99)	0.005	− 0.105	0.116
Breastfeeding	589	0.801	(0.19)	587	0.778	(0.19)	0.025**	0.003	0.046
Breastfeeding (standardized)	589	0.126	(0.98)	587	0.007	(0.99)	0.129**	0.016	0.242
Complementary feeding	589	0.382	(0.15)	587	0.344	(0.13)	0.038***	0.022	0.054
Complementary feeding (standardized)	589	0.306	(1.12)	587	0.020	(0.99)	0.287***	0.166	0.407
Benefits of animal products	589	0.358	(0.17)	587	0.372	(0.18)	− 0.014	− 0.034	0.006
Benefits of animal products (standardized)	589	− 0.038	(0.93)	587	0.039	(1.03)	− 0.080	− 0.192	0.032
Benefits of micronutrients	589	0.557	(0.16)	587	0.539	(0.17)	0.021**	0.002	0.039
Benefits of micronutrients (standardized)	589	0.153	(0.91)	587	0.048	(0.97)	0.120**	0.012	0.227
*Long term*									
Knowledge index	589	0.407	(0.14)	587	0.374	(0.13)	0.035***	0.020	0.050
Standardized knowledge index	589	0.251	(1.05)	587	− 0.001	(1.00)	0.269***	0.152	0.386
Diet during pregnancy	589	0.618	(0.19)	587	0.595	(0.20)	0.025**	0.003	0.047
Diet during pregnancy (standardized)	589	0.101	(0.96)	587	− 0.012	(1.00)	0.129**	0.017	0.240
Breastfeeding	589	0.747	(0.20)	587	0.735	(0.21)	0.014	− 0.009	0.038
Breastfeeding (standardized)	589	0.053	(0.93)	587	− 0.003	(1.00)	0.068	− 0.042	0.178
Complementary feeding	589	0.347	(0.15)	587	0.303	(0.14)	0.044***	0.028	0.060
Complementary feeding (standardized)	589	0.334	(1.09)	587	0.007	(1.00)	0.323***	0.203	0.443
Benefits of animal products	589	0.247	(0.25)	587	0.216	(0.24)	0.032**	0.004	0.061
Benefits of animal products (standardized)	589	0.121	(1.04)	587	− 0.005	(1.00)	0.132**	0.015	0.249
Benefits of micronutrients	589	0.421	(0.23)	587	0.386	(0.22)	0.040***	0.015	0.065
Benefits of micronutrients (standardized)	589	0.166	(1.04)	587	0.004	(1.00)	0.185***	0.069	0.301

Significant effects are in bold font; ***, **, and * indicate *p* < 0.01, *p* < 0.05, and *p* < 0.1, respectively. Adjusted regression controls for caregiver age (years), caregiver education (years), household size, monthly household per-capita income, and dummy variables for water connection, sewer connection, and bathroom or latrine. To ensure comparison of the same households over time, the sample is restricted to the balanced panel of households with complete knowledge information

Table 4 presents the ITT effects of the program on caregivers’ adoption of recommended IYCF practices. In the short term, the program increased adoption of recommended practices by about 0.248 SDs. However, these effects on practice dissipated in both groups over time, and the long-term effects of the program are less clear. We find a marginally significant effect of about 0.10 SD in the long term, but the effect does not hold under other sample and model specifications (Additional file 1: Tables S5 and S6).

**Table 4:** ITT effects on practice

	Treatment			Control			Adjusted		
	n	Mean	SD	n	Mean	SD	Effect	Lower bound	Upper bound
*Short term*									
Practice index	570	0.646	(0.13)	551	0.614	(0.13)	0.033***	0.017	0.048
Standardized practice index	570	0.221	(0.99)	551	− 0.020	(1.01)	0.248***	0.131	0.365
*Long term*									
Practice index	570	0.599	(0.17)	551	0.583	(0.17)	0.017*	− 0.003	0.037
Standardized practice index	570	0.088	(0.99)	551	− 0.006	(1.00)	0.100*	− 0.017	0.217

Significant effects are in bold font; ***, **, and * indicate p < 0.01, p < 0.05, and p < 0.1, respectively. Adjusted regression controls for caregiver age (years), caregiver education (years), household size, monthly household per-capita income, and dummy variables for water connection, sewer connection, and bathroom or latrine. To ensure comparison of the same households over time, the sample is restricted to the balanced panel of households with complete practice information

Heterogeneous ITT effects by caregiver characteristics, including age, number of children, baseline knowledge, education, and indigenous status, are presented in Table 5. ITT effects on caregiver knowledge were greater in the long term for caregivers with above median baseline knowledge (0.301 SDs). In the short term, caregivers above the median for age and number of children had an average increase of 0.343 SDs and 0.273 SDs in practices, respectively, compared to those below the median, but the effects on practices were not sustained in the long term. Additionally, non-indigenous caregivers had higher adoption of practices compared to indigenous caregivers in the long term (0.273 SDs). We found no significant effects by education. These results are consistent with inverse-probability weighted estimates and the full-sample results (Additional file 1: Tables S7 and S8). Associated graphs using the numerical version of these variables can be found in Additional file 1 (Additional file 1: Figs. S1–S8).

**Table 5:** Heterogeneous ITT effects, by caregiver characteristics

	Adjusted				
	n	Treatment	SD	Treatment* Panel	SD
***Panel 1. Age***					
*Short term*					
Knowledge index	1176	0.010	(0.01)	0.005	(0.01)
Standardized knowledge index	1176	0.105	(0.08)	0.053	(0.11)
Practice index	1121	0.011	(0.01)	0.045***	(0.02)
Standardized practice index	1121	0.081	(0.08)	0.343***	(0.12)
*Long term*					
Knowledge index	1176	0.023**	(0.01)	0.022	(0.02)
Standardized knowledge index	1176	0.180**	(0.08)	0.172	(0.12)
Practice index	1121	0.004	(0.01)	0.026	(0.02)
Standardized practice index	1121	0.026	(0.08)	0.155	(0.12)
***Panel 2. Number of children***					
*Short term*					
Knowledge index	1176	0.012	(0.01)	0.002	(0.01)
Standardized knowledge index	1176	0.123	(0.08)	0.020	(0.11)
Practice index	1121	0.017	(0.01)	0.036**	(0.02)
Standardized practice index	1121	0.128	(0.08)	0.273**	(0.12)
*Long term*					
Knowledge index	1176	0.029***	(0.01)	0.013	(0.02)
Standardized knowledge index	1176	0.223***	(0.08)	0.102	(0.12)
Practice index	1121	0.013	(0.01)	0.010	(0.02)
Standardized practice index	1121	0.075	(0.08)	0.057	(0.12)
***Panel 3. Baseline knowledge***					
*Short term*					
Knowledge index	1172	0.008	(0.01)	0.010	(0.01)
Standardized knowledge index	1172	0.084	(0.08)	0.096	(0.11)
Practice index	1117	0.021**	(0.01)	0.025	(0.02)
Standardized practice index	1117	0.162**	(0.08)	0.193	(0.12)
Knowledge index	1172	0.017*	(0.01)	0.039**	(0.02)
Standardized knowledge index	1172	0.130*	(0.08)	0.301**	(0.12)
Practice index	1117	0.009	(0.01)	0.017	(0.02)
Standardized practice index	1117	0.055	(0.08)	0.103	(0.12)
***Panel 4. Education***					
*Short term*					
Knowledge index	1176	0.018**	(0.01)	− 0.011	(0.01)
Standardized knowledge index	1176	0.177**	(0.08)	− 0.112	(0.11)
Practice index	1121	0.043***	(0.01)	− 0.023	(0.02)
Standardized practice index	1121	0.329***	(0.08)	− 0.172	(0.12)
*Long term*					
Knowledge index	1176	0.032***	(0.01)	0.002	(0.02)
Standardized knowledge index	1176	0.250***	(0.09)	0.015	(0.12)
Practice index	1121	0.019	(0.01)	− 0.007	(0.02)
Standardized practice index	1121	0.115	(0.08)	− 0.043	(0.12)
***Panel 5. Indigenous***					
*Short term*					
Knowledge index	1176	0.028**	(0.01)	− 0.018	(0.01)
Standardized knowledge index	1176	0.281**	(0.14)	− 0.179	(0.15)
Practice index	1121	0.019	(0.02)	0.016	(0.02)
Standardized practice index	1121	0.147	(0.13)	0.124	(0.15)
*Long term*					
Knowledge index	1176	0.043**	(0.02)	− 0.011	(0.02)
Standardized knowledge index	1176	0.336**	(0.15)	− 0.082	(0.16)
Practice index	1121	0.055**	(0.02)	− 0.046*	(0.03)
Standardized practice index	1121	0.326**	(0.15)	− 0.273*	(0.16)

Significant effects are in bold font; ***, **, and * indicate *p* < 0.01, *p* < 0.05, and *p* < 0.1, respectively. Regressions control for caregiver age (years), caregiver education (years), household size, monthly household per-capita income, and dummy variables for water connection, sewer connection, and bathroom or latrine. To ensure comparison of the same households over time, the sample is restricted to the balanced panel of households with complete knowledge/practice information

## Discussion

Reducing child malnutrition is a major development priority. Governments in Latin America are investing in strategies to improve child nutrition in the region, especially in high poverty and predominately indigenous areas, where rates of stunting and undernutrition are disproportionately high. This paper assesses the impacts of an entertainment-education intervention on caregivers’ IYCF knowledge and practices in a low-income, predominately indigenous district in El Alto, Bolivia. Our study builds upon a previous paper on the short-term effects of the program, expanding the analysis to include long-term and heterogeneous effects and focusing specifically on IYCF [26].

We find increases in caregivers’ knowledge of 0.134 and 0.269 SDs after 30 and 42 months, respectively. More specifically, we find caregivers who received the program had greater knowledge of breastfeeding, complementary feeding, and the benefits of micronutrients. These findings suggest that entertainment-education interventions are a promising mechanism for increasing IYCF knowledge and maintaining knowledge gains in the long term.

In addition to having positive effects on knowledge, the program also increased adherence to recommended IYCF practices in the short term by 0.248 SDs. However, over time, the effects on practices decreased and long-term effects are less certain. These results suggest that entertainment-education, while effective in promoting temporary adherence to IYCF recommendations, may not be sufficient for creating long-term, sustainable changes in practices.

While there is a large body of literature showing the effectiveness of various kinds of behavior-change interventions in modifying behavior, the evidence for sustained behavior change is much more limited. Very few studies assess long-term effects on behavior or address the issue of sustainability, and, many that do, find diminishing intervention effects over time [29]. Sustaining improvements in IYCF practices may require continued counseling and support services for participating families after the initial program ends, for example through community-based volunteers or mutually motivating peer support, use of cellphone reminders, or other behavior-change interventions yet to be evaluated. A study of a large-scale behavior-change communication intervention program to improve IYCF in Bangladesh assessed the sustainability of impacts after external funding from the initial donor agency ended. During the two years between the end of the program and the follow-up study, national partners, with the support of new donors, continued a limited-intensity version of the program, scaled up to 90% of the subdistricts in the country (compared to 10% initially). The authors find evidence for decreased but sustained impacts on IYCF practices after modifications [30]. This type of evidence suggests that entertainment-education followed by continued interpersonal counseling may be useful for sustaining improvements in IYCF practices, especially in a low-income or indigenous context.

Our study also finds that caregivers with above median baseline knowledge benefited the most from improvements in knowledge. Compared to those below the median, their overall IYCF knowledge was 0.301 SDs higher. In terms of practices, caregivers with above the median number of children and above median age saw the greatest improvements, suggesting those with greater experience raising children benefited the most. These results indicate that entertainment-education may be best used as an approach to complement and expand on previously accumulated knowledge and experience, rather than as a substitution mechanism to increase knowledge and practices among those with lower baseline rates.

We also find that, in the long term, adoption of practices was 0.273 SD lower for indigenous caregivers compared to non-indigenous caregivers. Given that the model controls for education and there is no heterogeneous effect of the program by education, we rule out differences in education as the underlying reason behind this result. Further evidence is needed to determine how to better tailor entertainment-education interventions to indigenous populations.

### Strengths and limitations

This study has important strengths. First, the experimental design with random assignment of households to treatment and control groups allows for credible estimates of the program’s effects on caregivers’ knowledge and practices. Additionally, households eligible for the study had children under 12 months at baseline, which is a critical window of opportunity for improving IYCF practices and, ultimately, child nutrition and growth.

A main limitation of the study is that the rate of sample attrition, by survey design and due to other uncontrollable factors, is high. Of the 2001 households originally surveyed, only 1225 (about 61%) two-parent households are included in all three rounds of data. However, attrition was found to be balanced across treatment and control groups, and additional methods were used to account for any selection bias due to attrition. A second limitation is potential spillovers. Although the intervention was limited to the confines of individual households, the project was carried out in a dense urban environment, where interactions between intervention and control populations could have been possible. To the extent that informational spillovers were positive, any such contamination would tend to downward bias estimated effect sizes.

## Conclusion

This study adds to the limited evidence on education and behavior-change interventions in Latin America and among indigenous populations. Our findings offer important policy implications. Education behavior-change interventions based on interactive play are a promising delivery model for improving and sustaining caregivers’ knowledge of IYCF recommendations. The findings of this study also suggest that entertainment-education by itself may not be sufficient to sustain improvements in IYCF practices, and that complementary approaches adapted to the cultural, educational, and experience profile of caregivers may be required to more permanently improve IYCF practices and ultimately child nutrition and growth.

### Supplementary Information

**Additional file 1: Appendix.** Supplementary tables and figures.

## Data Availability

All data and supporting material are publicly available at: http://dx.doi.org/10.18235/0001649.
