# Supplementary material for: Do improvements in infant and young child feeding (IYCF) practices endure the test of time?
Source: J Health Popul Nutr. 2024 Apr 5;43:49. doi: 10.1186/s41043-024-00507-5 (PMC10998429; doi:10.1186/s41043-024-00507-5)
Supplement: Supplementary file 1 — Additional file 1: Appendix. Supplementary tables and figures. [file 41043_2024_507_MOESM1_ESM.docx]

**Appendix for “Do improvements in infant and young child feeding (IYCF) practices endure the test of time?”**

**Solis Winters, Sebastian Martinez and Julia Johannsen**

**S1 File. Components in Knowledge Index**

1. Q1: Nutrition of the pregnant woman / quantity of food
2. Q2a: (3 correct answers) Nutrition of the pregnant woman / adequate foods
3. Q2b: (2 correct answers) Nutrition of the pregnant woman / adequate foods
4. Q2c: (1 correct answer) Nutrition of the pregnant woman / adequate foods
5. Q3: Nutrition of the pregnant woman / consumption of ferrous sulfate
6. Q4: Breastfeeding / time after birth
7. Q5: Breastfeeding / age for exclusive breastfeeding
8. Q6: Breastfeeding / hand washing
9. Q7: Complementary feeding / starting eating age
10. Q8: Complementary feeding / feed the baby if he/she brings things to his/her mouth
11. Q9: Breastfeeding / maximum age for breastfeeding
12. Q10: Complementary feeding / number of meals for a child
13. Q11: Complementary feeding / first food for a child
14. Q12a: (3 correct answers) Complementary feeding / benefits of thick foods
15. Q12b: (2 correct answers) Complementary feeding / benefits of thick foods
16. Q12b: (1 correct answer) Complementary feeding / benefits of thick foods
17. Q13: Minimum age for consuming protein of animal source / eggs
18. Q14: Minimum age for consuming protein of animal source / fish
19. Q15: Minimum age for consuming protein of animal source / meat
20. Q16: Minimum age for consuming protein of animal source / chicken
21. Q17a: (3 correct answers) Protein of animal source -organs / fish
22. Q17b: (2 correct answers) Protein of animal source - organs / fish
23. Q17c: (1 correct answer) Protein of animal source - organs / fish
24. Q18a: (3 correct answers) Protein of animal source -meat
25. Q18b: (2 correct answers) Protein of animal source -meat
26. Q18c: (1 correct answer) Protein of animal source -meat
27. Q19a: (3 correct answers) Protein of animal source -eggs
28. Q19b: (2 correct answers) Protein of animal source -eggs
29. Q19c: (1 correct answer) Protein of animal source -eggs
30. Q20a: (3 correct answers) Protein of animal source -chicken
31. Q20b: (2 correct answers) Protein of animal source -chicken
32. Q20c: (1 correct answer) Protein of animal source -chicken
33. Q21: Vitamin A- heard of
34. Q22: Vitamin A- frequency
35. Q23: Vitamin A - utility
36. Q24a: (3 correct answers) Anemia - how to prevent
37. Q24b: (2 correct answers) Anemia - how to prevent
38. Q24c: (1 correct answer) Anemia - how to prevent
39. Q25: Micro-nutrient powder (Chispitas) - how to consume
40. Q26a: (3 correct answers) Micro-nutrient powder (Chispitas) - what they are for
41. Q26b: (2 correct answers) Micro-nutrient powder (Chispitas) - what they are for
42. Q26c: (1 correct answer) Micro-nutrient powder (Chispitas) - what they are for
43. Q27: Nutritional supplement (Nutribebe) - what it is for

**S2 File. Components in Practice Index**

**Short Term**—

1. First time breastfed (20 missing)
2. Exclusive breastfeeding (4 missing)
3. Ever breastfed
4. Introduction of solid, semi-solid, or soft foods (52 missing)
5. Micro-nutrient powder (Chispitas) received
6. Micro-nutrient powder (Chispitas) consumed
7. Nutritional supplement (Nutribebe) received
8. Accompanies child during meals (11 missing)
9. Verbal stimulation (11 missing)
10. Additional stimulation (11 missing)
11. Positive actions against rejection (11 missing)
12. Minimum dietary diversity
13. Minimum dietary diversity (food score>=5) (6 missing)
14. Minimum dietary diversity (food score>=6) (6 missing)
15. Minimum dietary diversity (food score=7) (6 missing)
16. Consumption of iron-rich or iron-fortified food (5 missing)
17. No bottle feeding (4 missing)

**Long Term**—

1. Micro-nutrient powder (Chispitas) received (35 missing)
2. Micro-nutrient powder (Chispitas) consumed (35 missing)
3. Nutritional supplement (Nutribebe) received (35 missing)
4. Accompanies child during meals (9 missing)
5. Additional stimulation (44 missing)
6. Positive actions against rejection (44 missing)
7. Minimum dietary diversity (34 missing)
8. Minimum dietary diversity (food score>=5) (34 missing)
9. Minimum dietary diversity (food score>=6) (34 missing)
10. Minimum dietary diversity (food score=7) (34 missing)
11. Consumption of iron-rich or iron-fortified food (40 missing)

**S1 Table. Sample Attrition—full sample**

|  | **Treatment** | | **Control** | |  |  |  |
| --- | --- | --- | --- | --- | --- | --- | --- |
| **Attrition** | **Mean** | **SD** | **Mean** | **SD** | **Effect** | **Lower bound** | **Upper bound** |
| Baseline (n=2001) to Follow-Up 1 (n=1571) | 0.212 | (0.41) | 0.218 | (0.41) | -0.006 | -0.042 | 0.030 |
| Baseline (n=2001) to Follow-Up 2 (n=1236) | 0.378 | (0.49) | 0.387 | (0.49) | -0.009 | -0.051 | 0.034 |
| Follow-Up 1 (n=1582) to Follow-Up 2 (n=1234) | 0.214 | (0.41) | 0.226 | (0.42) | -0.012 | -0.053 | 0.029 |
| Total (n=2012) | 0.380 | (0.49) | 0.394 | (0.49) | -0.014 | -0.057 | 0.029 |
| Note. ***, **, and * indicate p<0.01, p<0.05, and p<0.1, respectively. | | | | | | | |

**S2 Table. Baseline Balance—attrited households (n=757)**

|  | **Treatment** | | **Control** | |  |  |  | |
| --- | --- | --- | --- | --- | --- | --- | --- | --- |
|  | **Mean** | **SD** | **Mean** | **SD** | **Effect** | **Lower bound** | | **Upper bound** |
| **Caregiver Characteristics** | n =370 | | n =387 | |  |  |  | |
| Gender (female=1) | 1.000 | (0.00) | 0.997 | (0.05) | 0.003 | -0.002 | 0.008 | |
| Age in years | 26.089 | (6.87) | 26.845 | (7.44) | -0.756 | -1.777 | 0.266 | |
| Self-identifies as indigenous | 0.792 | (0.41) | 0.811 | (0.39) | -0.019 | -0.076 | 0.038 | |
| Education in years | 9.105 | (3.47) | 8.884 | (3.82) | 0.222 | -0.298 | 0.741 | |
| Can read | 0.986 | (0.12) | 0.984 | (0.12) | 0.002 | -0.015 | 0.019 | |
| Worked in the past week | 0.314 | (0.46) | 0.357 | (0.48) | -0.043 | -0.110 | 0.024 | |
| Married or with partner | 0.841 | (0.37) | 0.829 | (0.38) | 0.011 | -0.042 | 0.064 | |
| Biological parent of target child | 0.951 | (0.22) | 0.933 | (0.25) | 0.019 | -0.015 | 0.052 | |
| Household head or spouse of head | 0.849 | (0.36) | 0.842 | (0.36) | 0.006 | -0.045 | 0.058 | |
|  |  |  |  |  |  |  |  | |
| **Spouse of Caregiver Characteristics** | n =305 | | n =315 | |  |  |  | |
| Gender (female=1) | 0.000 | (0.00) | 0.003 | (0.06) | -0.003 | -0.009 | 0.003 | |
| Age in years | 29.902 | (8.09) | 30.073 | (8.53) | -0.171 | -1.482 | 1.140 | |
| Self-identifies as indigenous | 0.810 | (0.39) | 0.829 | (0.38) | -0.019 | -0.080 | 0.042 | |
| Can read | 0.997 | (0.06) | 1.000 | (0.00) | -0.003 | -0.010 | 0.003 | |
| Education in years | 10.249 | (3.31) | 10.311 | (3.18) | -0.062 | -0.574 | 0.450 | |
| Worked in the past week | 0.964 | (0.19) | 0.959 | (0.20) | 0.005 | -0.025 | 0.036 | |
|  |  |  |  |  |  |  |  | |
| **Target Child Characteristics** | n =304 | | n =317 | |  |  |  | |
| Gender (female=1) | 0.477 | (0.50) | 0.486 | (0.50) | -0.009 | -0.084 | 0.066 | |
| Age in months | 6.171 | (4.00) | 6.666 | (3.92) | -0.495 | -1.119 | 0.130 | |
| Self-identifies as indigenous | 0.540 | (0.50) | 0.597 | (0.49) | -0.058 | -0.132 | 0.016 | |
|  |  |  |  |  |  |  |  | |

|  | **Treatment** | | | **Control** | |  |  | |  |
| --- | --- | --- | --- | --- | --- | --- | --- | --- | --- |
|  | **Mean** | **SD** | | **Mean** | **SD** | **Effect** | **Lower Bound** | **Upper Bound** | |
| **Household Characteristics** | n =370 | | | n =387 | |  |  | |  |
| Household size | 4.778 | | (1.97) | 4.685 | (1.90) | 0.094 | -0.183 | | 0.370 |
| Number of rooms | 2.014 | | (1.18) | 1.948 | (1.18) | 0.065 | -0.103 | | 0.234 |
| Monthly income per-capita (Bs) | 633.086 | | (613.23) | 596.848 | (471.71) | 36.238 | -42.072 | | 114.548 |
| Water=1 | 0.219 | | (0.41) | 0.243 | (0.43) | -0.024 | -0.084 | | 0.036 |
| Electricity=1 | 1.000 | | (0.00) | 0.987 | (0.11) | **0.013**** | 0.002 | | 0.024 |
| Home phone=1 | 0.043 | | (0.20) | 0.023 | (0.15) | 0.020 | -0.006 | | 0.046 |
| Cellphone=1 | 0.968 | | (0.18) | 0.966 | (0.18) | 0.001 | -0.024 | | 0.027 |
| Kitchen=1 | 1.000 | | (0.00) | 0.992 | (0.09) | **0.008*** | -0.001 | | 0.017 |
| Radio=1 | 0.808 | | (0.39) | 0.827 | (0.38) | -0.019 | -0.074 | | 0.036 |
| Television=1 | 0.959 | | (0.20) | 0.935 | (0.25) | 0.024 | -0.008 | | 0.056 |
| Refrigerator=1 | 0.165 | | (0.37) | 0.142 | (0.35) | 0.023 | -0.029 | | 0.074 |
| Vehicle=1 | 0.211 | | (0.41) | 0.183 | (0.39) | 0.027 | -0.030 | | 0.084 |
| Water pump=1 | 0.011 | | (0.10) | 0.003 | (0.05) | 0.008 | -0.003 | | 0.020 |
| Air conditioning=1 | 0.003 | | (0.05) | 0.000 | (0.00) | 0.003 | -0.003 | | 0.008 |
| Computer=1 | 0.119 | | (0.32) | 0.147 | (0.35) | -0.028 | -0.077 | | 0.020 |
| Bathroom or latrine=1 | 0.835 | | (0.37) | 0.817 | (0.39) | 0.019 | -0.036 | | 0.073 |
| Sewerage connection=1 | 0.343 | | (0.48) | 0.357 | (0.48) | -0.013 | -0.082 | | 0.055 |
| Note. ***, **, and * indicate p<0.01, p<0.05, and p<0.1, respectively. The sample is restricted to households that attrited, i.e. were not included in the first or second follow up. | | | | | | | | | |

**S3 Table. Effects on Knowledge – IPW estimates**

|  | **Treatment** | | | **Control** | | | **IPW** | | |
| --- | --- | --- | --- | --- | --- | --- | --- | --- | --- |
|  | **n** | **Mean** | **SD** | **n** | **Mean** | **SD** | **Effect** | **Lower bound** | **Upper bound** |
| ***Short Term*** |  |  |  |  |  |  |  |  |  |
| Knowledge Index | 589 | 0.486 | (0.09) | 587 | 0.473 | (0.10) | **0.014**** | 0.003 | 0.025 |
| Standardized Knowledge Index | 589 | 0.183 | (0.95) | 587 | 0.057 | (0.99) | **0.138**** | 0.026 | 0.249 |
|  |  |  |  |  |  |  |  |  |  |
| Diet During Pregnancy | 589 | 0.612 | (0.19) | 587 | 0.612 | (0.20) | 0.001 | -0.022 | 0.024 |
| Diet During Pregnancy (Standardized) | 589 | 0.036 | (0.95) | 587 | 0.034 | (0.99) | 0.006 | -0.109 | 0.120 |
|  |  |  |  |  |  |  |  |  |  |
| Breastfeeding | 589 | 0.801 | (0.19) | 587 | 0.778 | (0.19) | **0.027**** | 0.005 | 0.050 |
| Breastfeeding (Standardized) | 589 | 0.126 | (0.98) | 587 | 0.007 | (0.99) | **0.142**** | 0.024 | 0.259 |
|  |  |  |  |  |  |  |  |  |  |
| Complementary Feeding | 589 | 0.382 | (0.15) | 587 | 0.344 | (0.13) | **0.040***** | 0.023 | 0.057 |
| Complementary Feeding (Standardized) | 589 | 0.306 | (1.12) | 587 | 0.020 | (0.99) | **0.303***** | 0.177 | 0.430 |
|  |  |  |  |  |  |  |  |  |  |
| Benefits of Animal Products | 589 | 0.358 | (0.17) | 587 | 0.372 | (0.18) | -0.016 | -0.037 | 0.004 |
| Benefits of Animal Products (Standardized) | 589 | -0.038 | (0.93) | 587 | 0.039 | (1.03) | -0.092 | -0.207 | 0.023 |
|  |  |  |  |  |  |  |  |  |  |
| Benefits of Micronutrients | 589 | 0.557 | (0.16) | 587 | 0.539 | (0.17) | **0.021**** | 0.002 | 0.040 |
| Benefits of Micronutrients (Standardized) | 589 | 0.153 | (0.91) | 587 | 0.048 | (0.97) | **0.123**** | 0.013 | 0.234 |

|  | **Treatment** | | | **Control** | | | **IPW** | | |
| --- | --- | --- | --- | --- | --- | --- | --- | --- | --- |
|  | **n** | **Mean** | **SD** | **n** | **Mean** | **SD** | **Effect** | **Lower bound** | **Upper bound** |
| ***Long Term*** |  |  |  |  |  |  |  |  |  |
| Knowledge Index | 589 | 0.407 | (0.14) | 587 | 0.374 | (0.13) | **0.033***** | 0.017 | 0.049 |
| Standardized Knowledge Index | 589 | 0.251 | (1.05) | 587 | -0.001 | (1.00) | **0.254***** | 0.133 | 0.376 |
|  |  |  |  |  |  |  |  |  |  |
| Diet During Pregnancy | 589 | 0.618 | (0.19) | 587 | 0.595 | (0.20) | **0.022*** | -0.001 | 0.044 |
| Diet During Pregnancy (Standardized) | 589 | 0.101 | (0.96) | 587 | -0.012 | (1.00) | **0.110*** | -0.006 | 0.226 |
|  |  |  |  |  |  |  |  |  |  |
| Breastfeeding | 589 | 0.747 | (0.20) | 587 | 0.735 | (0.21) | **0.021*** | -0.003 | 0.046 |
| Breastfeeding (Standardized) | 589 | 0.053 | (0.93) | 587 | -0.003 | (1.00) | **0.100*** | -0.016 | 0.217 |
|  |  |  |  |  |  |  |  |  |  |
| Complementary Feeding | 589 | 0.347 | (0.15) | 587 | 0.303 | (0.14) | **0.047***** | 0.030 | 0.064 |
| Complementary Feeding (Standardized) | 589 | 0.334 | (1.09) | 587 | 0.007 | (1.00) | **0.346***** | 0.221 | 0.470 |
|  |  |  |  |  |  |  |  |  |  |
| Benefits of Animal Products | 589 | 0.247 | (0.25) | 587 | 0.216 | (0.24) | **0.026*** | -0.004 | 0.055 |
| Benefits of Animal Products (Standardized) | 589 | 0.121 | (1.04) | 587 | -0.005 | (1.00) | **0.105*** | -0.015 | 0.225 |
|  |  |  |  |  |  |  |  |  |  |
| Benefits of Micronutrients | 589 | 0.421 | (0.23) | 587 | 0.386 | (0.22) | **0.036***** | 0.010 | 0.062 |
| Benefits of Micronutrients (Standardized) | 589 | 0.166 | (1.04) | 587 | 0.004 | (1.00) | **0.165***** | 0.045 | 0.286 |
| Note. ***, **, and * indicate p<0.01, p<0.05, and p<0.1, respectively. Regressions control for caregiver age (years), caregiver education (years), household size, monthly household per-capita income, and dummy variables for water connection, sewer connection, and bathroom or latrine. Inverse-probability weights (IPW) are also included. To ensure comparison of the same households over time, the sample is restricted to the balanced panel of households with complete knowledge information. | | | | | | | | | |

**S4 Table. Effects on Knowledge – full sample**

|  | **Treatment** | | | **Control** | | | **Adjusted** | | | **IPW** | | |
| --- | --- | --- | --- | --- | --- | --- | --- | --- | --- | --- | --- | --- |
|  | **n** | **Mean** | **SD** | **n** | **Mean** | **SD** | **Effect** | **Lower bound** | **Upper bound** | **Effect** | **Lower bound** | **Upper bound** |
| ***Short Term*** |  |  |  |  |  |  |  |  |  |  |  |  |
| Knowledge Index | 788 | 0.484 | (0.10) | 784 | 0.468 | (0.10) | **0.017***** | 0.007 | 0.027 | **0.018***** | 0.007 | 0.030 |
| Standardized Knowledge Index | 788 | 0.168 | (0.99) | 784 | -0.000 | (1.00) | **0.173***** | 0.075 | 0.271 | **0.182***** | 0.067 | 0.297 |
|  |  |  |  |  |  |  |  |  |  |  |  |  |
| Diet During Pregnancy | 788 | 0.609 | (0.20) | 784 | 0.605 | (0.20) | 0.003 | -0.016 | 0.023 | 0.007 | -0.015 | 0.029 |
| Diet During Pregnancy (Standardized) | 788 | 0.020 | (0.96) | 784 | -0.000 | (1.00) | 0.017 | -0.080 | 0.114 | 0.033 | -0.073 | 0.140 |
|  |  |  |  |  |  |  |  |  |  |  |  |  |
| Breastfeeding | 788 | 0.794 | (0.19) | 784 | 0.777 | (0.19) | **0.019*** | -0.000 | 0.038 | 0.015 | -0.007 | 0.036 |
| Breastfeeding (Standardized) | 788 | 0.091 | (1.00) | 784 | 0.000 | (1.00) | **0.098*** | -0.002 | 0.197 | 0.076 | -0.034 | 0.187 |
|  |  |  |  |  |  |  |  |  |  |  |  |  |
| Complementary Feeding | 788 | 0.379 | (0.15) | 784 | 0.342 | (0.13) | **0.037***** | 0.023 | 0.050 | **0.037***** | 0.021 | 0.052 |
| Complementary Feeding (Standardized) | 788 | 0.279 | (1.11) | 784 | -0.000 | (1.00) | **0.278***** | 0.173 | 0.383 | **0.280***** | 0.162 | 0.398 |
|  |  |  |  |  |  |  |  |  |  |  |  |  |
| Benefits of Animal Products | 788 | 0.363 | (0.17) | 784 | 0.365 | (0.18) | -0.002 | -0.019 | 0.016 | 0.000 | -0.019 | 0.019 |
| Benefits of Animal Products (Standardized) | 788 | -0.010 | (0.94) | 784 | -0.000 | (1.00) | -0.009 | -0.106 | 0.088 | 0.001 | -0.104 | 0.106 |
|  |  |  |  |  |  |  |  |  |  |  |  |  |
| Benefits of Micronutrients | 788 | 0.554 | (0.16) | 784 | 0.531 | (0.17) | **0.024***** | 0.008 | 0.041 | **0.025***** | 0.006 | 0.045 |
| Benefits of Micronutrients (Standardized) | 788 | 0.131 | (0.95) | 784 | 0.000 | (1.00) | **0.140***** | 0.044 | 0.236 | **0.147***** | 0.037 | 0.258 |

|  | **Treatment** | | | **Control** | | | **Adjusted** | | | **IPW** | | |
| --- | --- | --- | --- | --- | --- | --- | --- | --- | --- | --- | --- | --- |
|  | **n** | **Mean** | **SD** | **n** | **Mean** | **SD** | **Effect** | **Lower bound** | **Upper bound** | **Effect** | **Lower bound** | **Upper bound** |
| ***Long Term*** |  |  |  |  |  |  |  |  |  |  |  |  |
| Knowledge Index | 589 | 0.407 | (0.14) | 587 | 0.374 | (0.13) | **0.035***** | 0.020 | 0.050 | **0.033***** | 0.017 | 0.049 |
| Standardized Knowledge Index | 589 | 0.251 | (1.05) | 587 | -0.001 | (1.00) | **0.269***** | 0.152 | 0.386 | **0.254***** | 0.133 | 0.376 |
|  |  |  |  |  |  |  |  |  |  |  |  |  |
| Diet During Pregnancy | 589 | 0.618 | (0.19) | 587 | 0.595 | (0.20) | **0.025**** | 0.003 | 0.047 | **0.022*** | -0.001 | 0.044 |
| Diet During Pregnancy (Standardized) | 589 | 0.101 | (0.96) | 587 | -0.012 | (1.00) | **0.129**** | 0.017 | 0.240 | **0.110*** | -0.006 | 0.226 |
|  |  |  |  |  |  |  |  |  |  |  |  |  |
| Breastfeeding | 589 | 0.747 | (0.20) | 587 | 0.735 | (0.21) | 0.014 | -0.009 | 0.038 | **0.021*** | -0.003 | 0.046 |
| Breastfeeding (Standardized) | 589 | 0.053 | (0.93) | 587 | -0.003 | (1.00) | 0.068 | -0.042 | 0.178 | **0.100*** | -0.016 | 0.217 |
|  |  |  |  |  |  |  |  |  |  |  |  |  |
| Complementary Feeding | 589 | 0.347 | (0.15) | 587 | 0.303 | (0.14) | **0.044***** | 0.028 | 0.060 | **0.047***** | 0.030 | 0.064 |
| Complementary Feeding (Standardized) | 589 | 0.334 | (1.09) | 587 | 0.007 | (1.00) | **0.323***** | 0.203 | 0.443 | **0.346***** | 0.221 | 0.470 |
|  |  |  |  |  |  |  |  |  |  |  |  |  |
| Benefits of Animal Products | 589 | 0.247 | (0.25) | 587 | 0.216 | (0.24) | **0.032**** | 0.004 | 0.061 | **0.026*** | -0.004 | 0.055 |
| Benefits of Animal Products (Standardized) | 589 | 0.121 | (1.04) | 587 | -0.005 | (1.00) | **0.132**** | 0.015 | 0.249 | **0.105*** | -0.015 | 0.225 |
|  |  |  |  |  |  |  |  |  |  |  |  |  |
| Benefits of Micronutrients | 589 | 0.421 | (0.23) | 587 | 0.386 | (0.22) | **0.040***** | 0.015 | 0.065 | **0.036***** | 0.010 | 0.062 |
| Benefits of Micronutrients (Standardized) | 589 | 0.166 | (1.04) | 587 | 0.004 | (1.00) | **0.185***** | 0.069 | 0.301 | **0.165***** | 0.045 | 0.286 |
| Note. ***, **, and * indicate p<0.01, p<0.05, and p<0.1, respectively. Regressions control for caregiver age (years), caregiver education (years), household size, monthly household per-capita income, and dummy variables for water connection, sewer connection, and bathroom or latrine. Models with inverse-probability weights (IPW) are also included. | | | | | | | | | | | | |

**S5 Table. Effects on Practice – IPW estimates**

|  | **Treatment** | | | **Control** | | | **IPW** | | |
| --- | --- | --- | --- | --- | --- | --- | --- | --- | --- |
|  | **n** | **Mean** | **SD** | **n** | **Mean** | **SD** | **Effect** | **Lower bound** | **Upper bound** |
| ***Short Term*** |  |  |  |  |  |  |  |  |  |
| Practice Index | 570 | 0.646 | (0.13) | 551 | 0.614 | (0.13) | **0.032***** | 0.015 | 0.048 |
| Standardized Practice Index | 570 | 0.221 | (0.99) | 551 | -0.020 | (1.01) | **0.240***** | 0.117 | 0.363 |
|  |  |  |  |  |  |  |  |  |  |
| ***Long Term*** |  |  |  |  |  |  |  |  |  |
| Practice Index | 570 | 0.599 | (0.17) | 551 | 0.583 | (0.17) | 0.013 | -0.008 | 0.035 |
| Standardized Practice Index | 570 | 0.088 | (0.99) | 551 | -0.006 | (1.00) | 0.078 | -0.048 | 0.205 |
| Note. ***, **, and * indicate p<0.01, p<0.05, and p<0.1, respectively. Regressions control for caregiver age (years), caregiver education (years), household size, monthly household per-capita income, and dummy variables for water connection, sewer connection, and bathroom or latrine. Inverse-probability weights (IPW) are also included. To ensure comparison of the same households over time, the sample is restricted to the balanced panel of households with complete practice information. | | | | | | | | | |

**S6 Table. Effects on Practice – full sample**

|  | **Treatment** | | | **Control** | | | **Adjusted** | | | **IPW** | | |
| --- | --- | --- | --- | --- | --- | --- | --- | --- | --- | --- | --- | --- |
|  | **n** | **Mean** | **SD** | **n** | **Mean** | **SD** | **Effect** | **Lower bound** | **Upper bound** | **Effect** | **Lower bound** | **Upper bound** |
| ***Short Term*** |  |  |  |  |  |  |  |  |  |  |  |  |
| Practice Index | 753 | 0.642 | (0.13) | 743 | 0.616 | (0.13) | **0.027***** | 0.014 | 0.040 | **0.029***** | 0.013 | 0.045 |
| Standardized Practice Index | 753 | 0.197 | (0.99) | 743 | 0.000 | (1.00) | **0.204***** | 0.102 | 0.305 | **0.219***** | 0.100 | 0.339 |
|  |  |  |  |  |  |  |  |  |  |  |  |  |
| ***Long Term*** |  |  |  |  |  |  |  |  |  |  |  |  |
| Practice Index | 602 | 0.598 | (0.17) | 594 | 0.584 | (0.17) | 0.015 | -0.004 | 0.034 | 0.010 | -0.010 | 0.031 |
| Standardized Practice Index | 602 | 0.083 | (0.98) | 594 | -0.000 | (1.00) | 0.086 | -0.027 | 0.200 | 0.062 | -0.061 | 0.185 |
| Note. ***, **, and * indicate p<0.01, p<0.05, and p<0.1, respectively. Regressions control for caregiver age (years), caregiver education (years), household size, monthly household per-capita income, and dummy variables for water connection, sewer connection, and bathroom or latrine. Models with inverse-probability weights (IPW) are also included. | | | | | | | | | | | | |

**S7 Table. Heterogeneous Effects – IPW estimates**

|  | **IPW** | | | | |
| --- | --- | --- | --- | --- | --- |
|  | **n** | **Treatment** | **SD** | **Treatment* Panel** | **SD** |
| **Panel 1. Age** |  |  |  |  |  |
| ***Short Term*** |  |  |  |  |  |
| Knowledge Index | 1176 | 0.010 | (0.01) | 0.007 | (0.01) |
| Standardized Knowledge Index | 1176 | 0.103 | (0.08) | 0.068 | (0.11) |
| Practice Index | 1121 | 0.010 | (0.01) | **0.047***** | (0.02) |
| Standardized Practice Index | 1121 | 0.076 | (0.09) | **0.357***** | (0.13) |
|  |  |  |  |  |  |
| ***Long Term*** |  |  |  |  |  |
| Knowledge Index | 1176 | **0.019*** | (0.01) | **0.029*** | (0.02) |
| Standardized Knowledge Index | 1176 | **0.145*** | (0.09) | **0.228*** | (0.12) |
| Practice Index | 1121 | -0.005 | (0.02) | **0.040*** | (0.02) |
| Standardized Practice Index | 1121 | -0.029 | (0.09) | **0.240*** | (0.13) |
|  |  |  |  |  |  |
| **Panel 2. Number of Children** |  |  |  |  |  |
| ***Short Term*** |  |  |  |  |  |
| Knowledge Index | 1176 | 0.012 | (0.01) | 0.005 | (0.01) |
| Standardized Knowledge Index | 1176 | 0.116 | (0.08) | 0.047 | (0.11) |
| Practice Index | 1121 | 0.015 | (0.01) | **0.040**** | (0.02) |
| Standardized Practice Index | 1121 | 0.112 | (0.08) | **0.306**** | (0.13) |
|  |  |  |  |  |  |
| ***Long Term*** |  |  |  |  |  |
| Knowledge Index | 1176 | **0.024**** | (0.01) | 0.020 | (0.02) |
| Standardized Knowledge Index | 1176 | **0.189**** | (0.08) | 0.156 | (0.13) |
| Practice Index | 1121 | 0.005 | (0.01) | 0.020 | (0.02) |
| Standardized Practice Index | 1121 | 0.032 | (0.09) | 0.117 | (0.13) |
|  |  |  |  |  |  |
| **Panel 3. Baseline Knowledge** |  |  |  |  |  |
| ***Short Term*** |  |  |  |  |  |
| Knowledge Index | 1172 | 0.009 | (0.01) | 0.010 | (0.01) |
| Standardized Knowledge Index | 1172 | 0.089 | (0.08) | 0.096 | (0.11) |
| Practice Index | 1117 | 0.018 | (0.01) | **0.030*** | (0.02) |
| Standardized Practice Index | 1117 | 0.138 | (0.09) | **0.230*** | (0.12) |
|  |  |  |  |  |  |
| ***Long Term*** |  |  |  |  |  |
| Knowledge Index | 1172 | 0.012 | (0.01) | **0.045***** | (0.02) |
| Standardized Knowledge Index | 1172 | 0.095 | (0.08) | **0.351***** | (0.12) |
| Practice Index | 1117 | 0.003 | (0.02) | 0.024 | (0.02) |
| Standardized Practice Index | 1117 | 0.018 | (0.09) | 0.140 | (0.13) |

|  | **IPW** | | | | |
| --- | --- | --- | --- | --- | --- |
|  | **n** | **Treatment** | **SD** | **Treatment* Panel** | **SD** |
| **Panel 4. Education** |  |  |  |  |  |
| ***Short Term*** |  |  |  |  |  |
| Knowledge Index | 1176 | **0.018**** | (0.01) | -0.012 | (0.01) |
| Standardized Knowledge Index | 1176 | **0.185**** | (0.08) | -0.118 | (0.11) |
| Practice Index | 1121 | **0.040***** | (0.01) | -0.017 | (0.02) |
| Standardized Practice Index | 1121 | **0.299***** | (0.09) | -0.131 | (0.13) |
|  |  |  |  |  |  |
| ***Long Term*** |  |  |  |  |  |
| Knowledge Index | 1176 | **0.028**** | (0.01) | 0.007 | (0.02) |
| Standardized Knowledge Index | 1176 | **0.219**** | (0.09) | 0.054 | (0.12) |
| Practice Index | 1121 | 0.012 | (0.02) | 0.001 | (0.02) |
| Standardized Practice Index | 1121 | 0.070 | (0.09) | 0.005 | (0.13) |
|  |  |  |  |  |  |
| **Panel 5. Indigenous** |  |  |  |  |  |
| ***Short Term*** |  |  |  |  |  |
| Knowledge Index | 1176 | **0.026*** | (0.01) | -0.015 | (0.01) |
| Standardized Knowledge Index | 1176 | **0.259*** | (0.14) | -0.149 | (0.15) |
| Practice Index | 1121 | 0.020 | (0.02) | 0.014 | (0.02) |
| Standardized Practice Index | 1121 | 0.153 | (0.14) | 0.109 | (0.15) |
|  |  |  |  |  |  |
| ***Long Term*** |  |  |  |  |  |
| Knowledge Index | 1176 | **0.036*** | (0.02) | -0.004 | (0.02) |
| Standardized Knowledge Index | 1176 | **0.279*** | (0.15) | -0.031 | (0.16) |
| Practice Index | 1121 | **0.054**** | (0.03) | **-0.050*** | (0.03) |
| Standardized Practice Index | 1121 | **0.321**** | (0.15) | **-0.297*** | (0.17) |
| Note. ***, **, and * indicate p<0.01, p<0.05, and p<0.1, respectively. Regressions control for caregiver age (years), caregiver education (years), household size, monthly household per-capita income, and dummy variables for water connection, sewer connection, and bathroom or latrine. Inverse-probability weights (IPW) are also included. To ensure comparison of the same households over time, the sample is restricted to the balanced panel of households with complete knowledge/practice information. | | | | | |

**S8 Table. Heterogeneous ITT effects—full sample**

|  | **Adjusted** | | | | |  | **IPW** | | | |
| --- | --- | --- | --- | --- | --- | --- | --- | --- | --- | --- |
|  | **n** | **Treatment** | **SD** | **Treatment* Panel** | **SD** |  | **Treatment** | **SD** | **Treatment* Panel** | **SD** |
| **Panel 1. Age** |  |  |  |  |  |  |  |  |  |  |
| ***Short Term*** |  |  |  |  |  |  |  |  |  |  |
| Knowledge Index | 1561 | 0.011 | (0.01) | 0.013 | (0.01) |  | 0.010 | (0.01) | **0.019*** | (0.01) |
| Standardized Knowledge Index | 1561 | 0.108 | (0.07) | 0.133 | (0.10) |  | 0.097 | (0.08) | **0.195*** | (0.11) |
| Practice Index | 1487 | 0.012 | (0.01) | **0.031**** | (0.01) |  | 0.017 | (0.01) | **0.028*** | (0.02) |
| Standardized Practice Index | 1487 | 0.094 | (0.07) | **0.233**** | (0.10) |  | 0.128 | (0.09) | **0.210*** | (0.12) |
|  |  |  |  |  |  |  |  |  |  |  |
| ***Long Term*** |  |  |  |  |  |  |  |  |  |  |
| Knowledge Index | 1196 | **0.024**** | (0.01) | 0.021 | (0.02) |  | **0.019*** | (0.01) | **0.029*** | (0.02) |
| Standardized Knowledge Index | 1196 | **0.184**** | (0.08) | 0.166 | (0.12) |  | **0.146*** | (0.08) | **0.222*** | (0.12) |
| Practice Index | 1188 | 0.003 | (0.01) | 0.023 | (0.02) |  | -0.008 | (0.02) | **0.040*** | (0.02) |
| Standardized Practice Index | 1188 | 0.020 | (0.08) | 0.139 | (0.12) |  | -0.045 | (0.09) | **0.238*** | (0.12) |
|  |  |  |  |  |  |  |  |  |  |  |
| **Panel 2. Number of Children** |  |  |  |  |  |  |  |  |  |  |
| ***Short Term*** |  |  |  |  |  |  |  |  |  |  |
| Knowledge Index | 1561 | **0.015**** | (0.01) | 0.005 | (0.01) |  | 0.011 | (0.01) | **0.020*** | (0.01) |
| Standardized Knowledge Index | 1561 | **0.153**** | (0.07) | 0.046 | (0.10) |  | 0.107 | (0.07) | **0.201*** | (0.12) |
| Practice Index | 1487 | 0.014 | (0.01) | **0.031**** | (0.01) |  | 0.011 | (0.01) | **0.047***** | (0.02) |
| Standardized Practice Index | 1487 | 0.104 | (0.07) | **0.237**** | (0.11) |  | 0.086 | (0.07) | **0.355***** | (0.13) |
|  |  |  |  |  |  |  |  |  |  |  |
| ***Long Term*** |  |  |  |  |  |  |  |  |  |  |
| Knowledge Index | 1196 | **0.029***** | (0.01) | 0.014 | (0.02) |  | **0.024**** | (0.01) | 0.021 | (0.02) |
| Standardized Knowledge Index | 1196 | **0.222***** | (0.08) | 0.104 | (0.12) |  | **0.186**** | (0.08) | 0.159 | (0.12) |
| Practice Index | 1188 | 0.009 | (0.01) | 0.013 | (0.02) |  | -0.000 | (0.01) | 0.026 | (0.02) |
| Standardized Practice Index | 1188 | 0.051 | (0.08) | 0.079 | (0.12) |  | -0.000 | (0.09) | 0.152 | (0.12) |

|  | **Adjusted** | | | | |  | **IPW** | | | |
| --- | --- | --- | --- | --- | --- | --- | --- | --- | --- | --- |
|  | **n** | **Treatment** | **SD** | **Treatment* Panel** | **SD** |  | **Treatment** | **SD** | **Treatment* Panel** | **SD** |
| **Panel 3. Baseline Knowledge** |  |  |  |  |  |  |  |  |  |  |
| ***Short Term*** |  |  |  |  |  |  |  |  |  |  |
| Knowledge Index | 1557 | 0.009 | (0.01) | 0.018* | (0.01) |  | 0.013* | (0.01) | 0.010 | (0.01) |
| Standardized Knowledge Index | 1557 | 0.087 | (0.07) | 0.185* | (0.10) |  | 0.135* | (0.08) | 0.096 | (0.12) |
| Practice Index | 1483 | **0.014** | (0.01) | 0.028** | (0.01) |  | 0.018 | (0.01) | 0.025 | (0.02) |
| Standardized Practice Index | 1483 | **0.109** | (0.07) | 0.214** | (0.10) |  | 0.134 | (0.09) | 0.188 | (0.12) |
|  |  |  |  |  |  |  |  |  |  |  |
| ***Long Term*** |  |  |  |  |  |  |  |  |  |  |
| Knowledge Index | 1192 | **0.018*** | (0.01) | **0.037**** | (0.02) |  | 0.013 | (0.01) | **0.043***** | (0.02) |
| Standardized Knowledge Index | 1192 | **0.140*** | (0.08) | **0.283**** | (0.12) |  | 0.103 | (0.08) | **0.333***** | (0.12) |
| Practice Index | 1184 | 0.006 | (0.01) | 0.019 | (0.02) |  | 0.001 | (0.01) | 0.022 | (0.02) |
| Standardized Practice Index | 1184 | 0.038 | (0.08) | 0.110 | (0.12) |  | 0.004 | (0.09) | 0.133 | (0.12) |
|  |  |  |  |  |  |  |  |  |  |  |
| **Panel 4. Education** |  |  |  |  |  |  |  |  |  |  |
| ***Short Term*** |  |  |  |  |  |  |  |  |  |  |
| Knowledge Index | 1561 | **0.022***** | (0.01) | -0.011 | (0.01) |  | **0.027***** | (0.01) | **-0.019*** | (0.01) |
| Standardized Knowledge Index | 1561 | **0.222***** | (0.07) | -0.115 | (0.10) |  | **0.267***** | (0.09) | **-0.194*** | (0.12) |
| Practice Index | 1487 | **0.034***** | (0.01) | -0.015 | (0.01) |  | **0.036***** | (0.01) | -0.017 | (0.02) |
| Standardized Practice Index | 1487 | **0.255***** | (0.07) | -0.112 | (0.10) |  | **0.276***** | (0.09) | -0.126 | (0.12) |
|  |  |  |  |  |  |  |  |  |  |  |
| ***Long Term*** |  |  |  |  |  |  |  |  |  |  |
| Knowledge Index | 1196 | **0.032***** | (0.01) | 0.002 | (0.02) |  | **0.028**** | (0.01) | 0.006 | (0.02) |
| Standardized Knowledge Index | 1196 | **0.250***** | (0.08) | 0.014 | (0.12) |  | **0.218**** | (0.09) | 0.050 | (0.12) |
| Practice Index | 1188 | 0.015 | (0.01) | -0.004 | (0.02) |  | 0.007 | (0.02) | 0.005 | (0.02) |
| Standardized Practice Index | 1188 | 0.090 | (0.08) | -0.021 | (0.11) |  | 0.043 | (0.09) | 0.029 | (0.13) |

|  | **Adjusted** | | | | |  | **IPW** | | | |
| --- | --- | --- | --- | --- | --- | --- | --- | --- | --- | --- |
|  | **n** | **Treatment** | **SD** | **Treatment* Panel** | **SD** |  | **Treatment** | **SD** | **Treatment* Panel** | **SD** |
| **Panel 5. Indigenous** |  |  |  |  |  |  |  |  |  |  |
| ***Short Term*** |  |  |  |  |  |  |  |  |  |  |
| Knowledge Index | 1561 | **0.032***** | (0.01) | -0.018 | (0.01) |  | **0.035**** | (0.02) | -0.021 | (0.02) |
| Standardized Knowledge Index | 1561 | **0.318***** | (0.12) | -0.176 | (0.13) |  | **0.353**** | (0.15) | -0.213 | (0.16) |
| Practice Index | 1487 | 0.019 | (0.02) | 0.009 | (0.02) |  | 0.036* | (0.02) | -0.010 | (0.02) |
| Standardized Practice Index | 1487 | 0.147 | (0.12) | 0.069 | (0.13) |  | 0.276* | (0.17) | -0.072 | (0.17) |
|  |  |  |  |  |  |  |  |  |  |  |
| ***Long Term*** |  |  |  |  |  |  |  |  |  |  |
| Knowledge Index | 1196 | **0.044**** | (0.02) | -0.011 | (0.02) |  | **0.036*** | (0.02) | -0.005 | (0.02) |
| Standardized Knowledge Index | 1196 | **0.338**** | (0.15) | -0.083 | (0.16) |  | **0.281*** | (0.15) | -0.035 | (0.16) |
| Practice Index | 1188 | **0.051**** | (0.02) | **-0.043*** | (0.03) |  | **0.048*** | (0.02) | **-0.045*** | (0.03) |
| Standardized Practice Index | 1188 | **0.300**** | (0.14) | **-0.258*** | (0.16) |  | **0.282*** | (0.15) | **-0.269*** | (0.16) |
| Note. ***, **, and * indicate p<0.01, p<0.05, and p<0.1, respectively. Regressions control for caregiver age (years), caregiver education (years), household size, monthly household per-capita income, and dummy variables for water connection, sewer connection, and bathroom or latrine. Models with inverse-probability weights (IPW) are also included. | | | | | | | | | | |

**S1 Figure. Short-term Effects by Age**


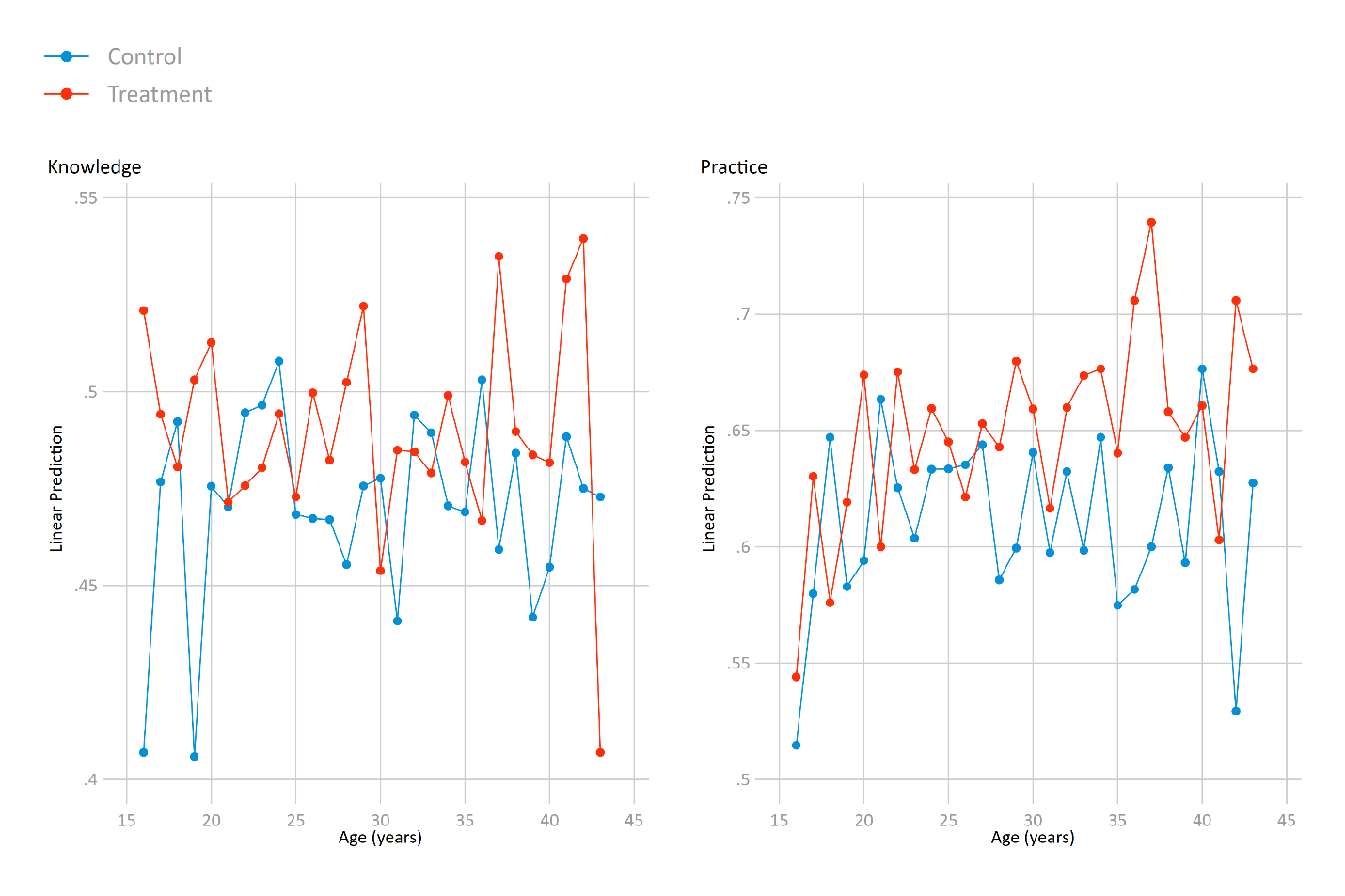


**S2 Figure. Long-term Effects by Age**

**
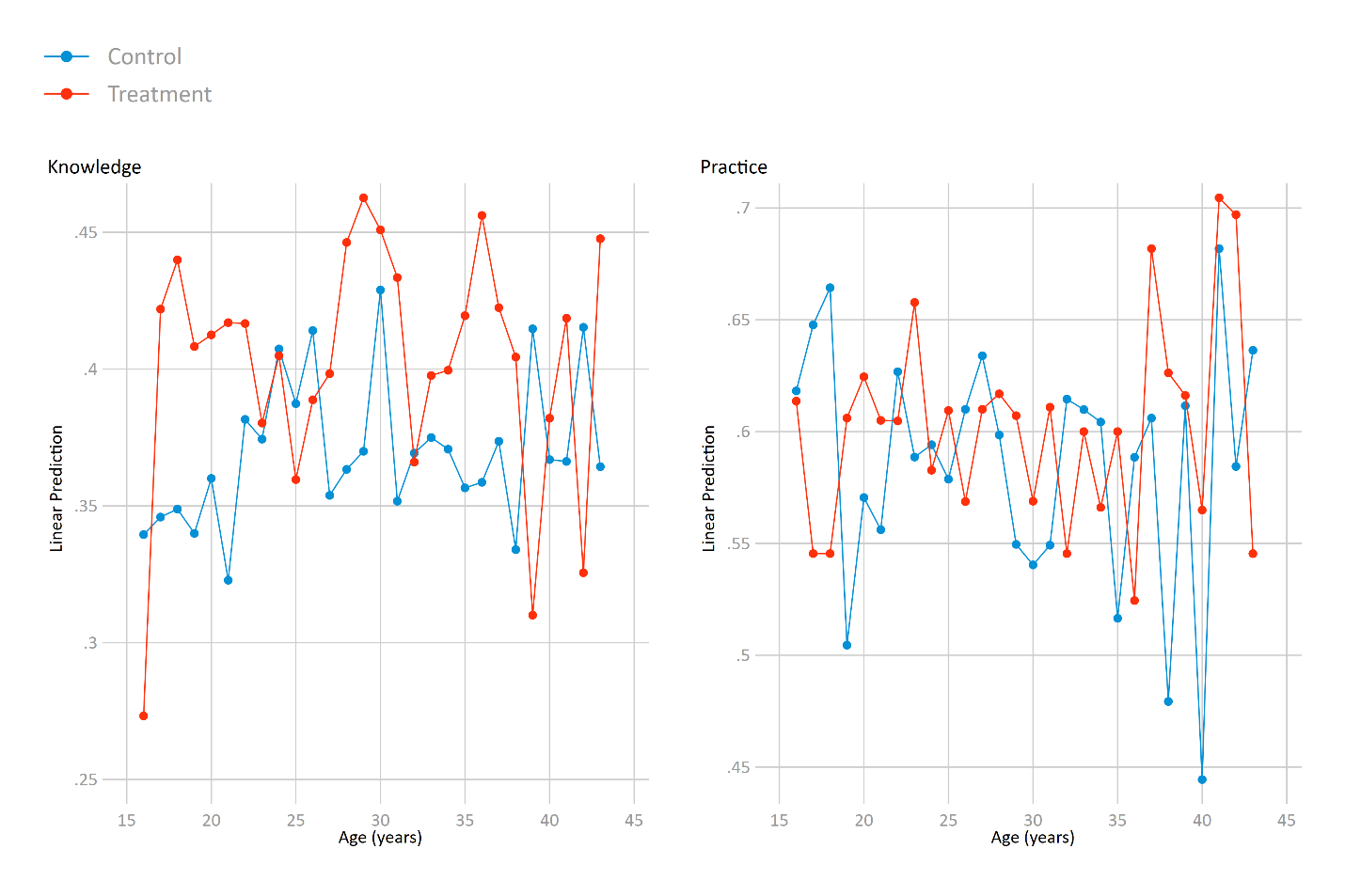
**

**S3 Figure. Short-term Effects by Number of Children**


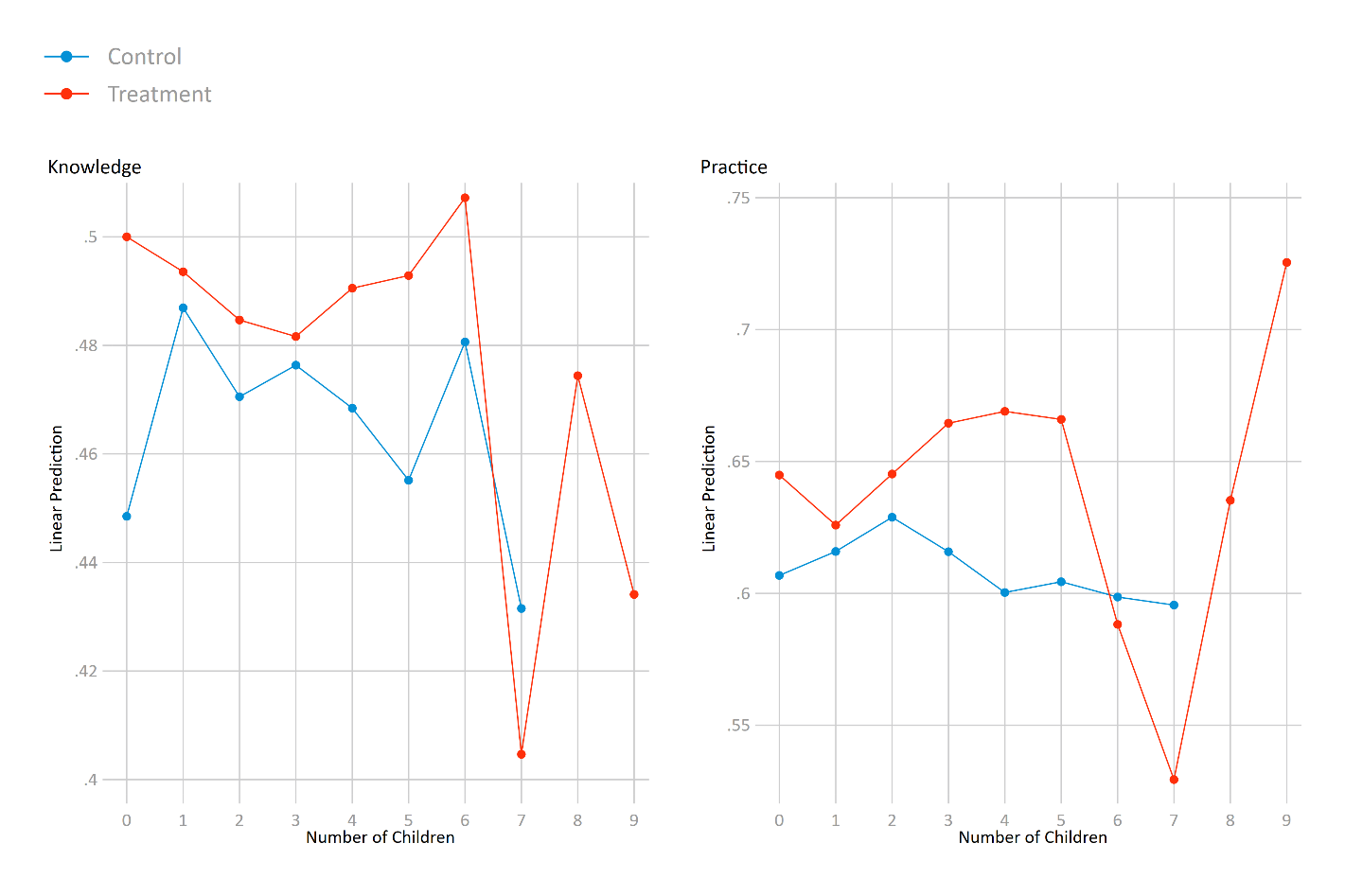


**S4 Figure. Long-term Effects by Number of Children**

**
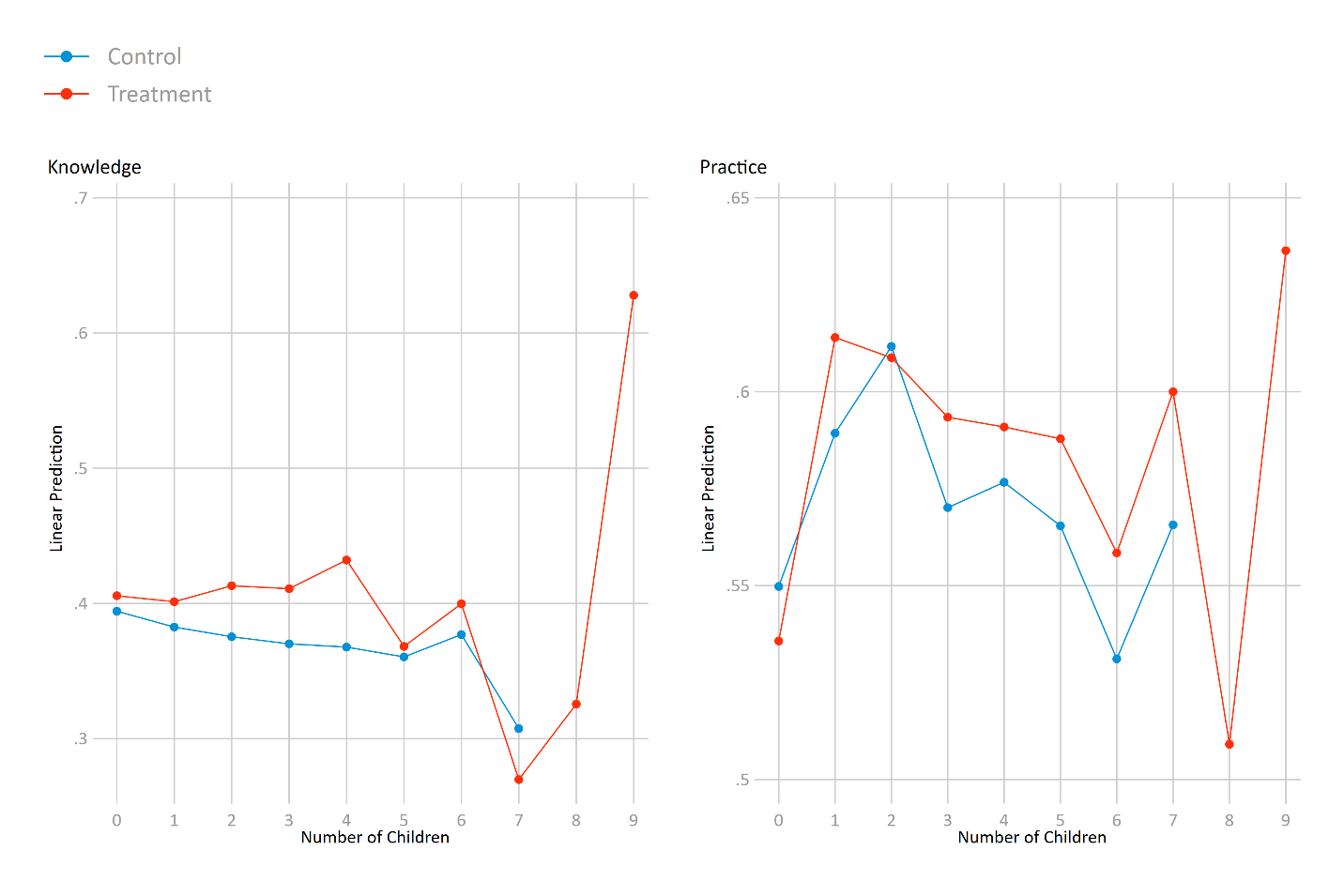
**

**S5 Figure. Short-term Effects by Baseline Knowledge**


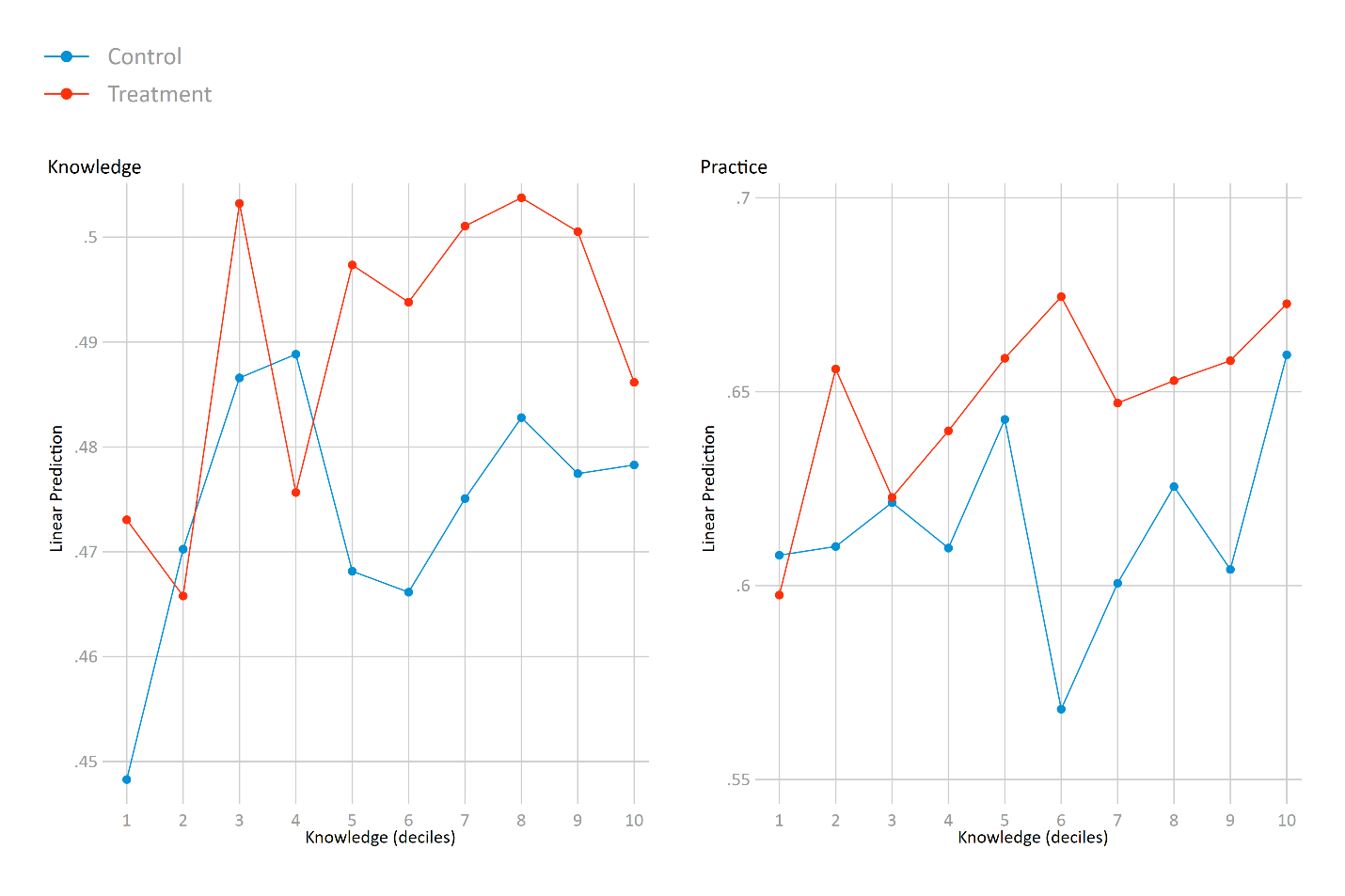


**S6 Figure. Long-term Effects by Baseline Knowledge**


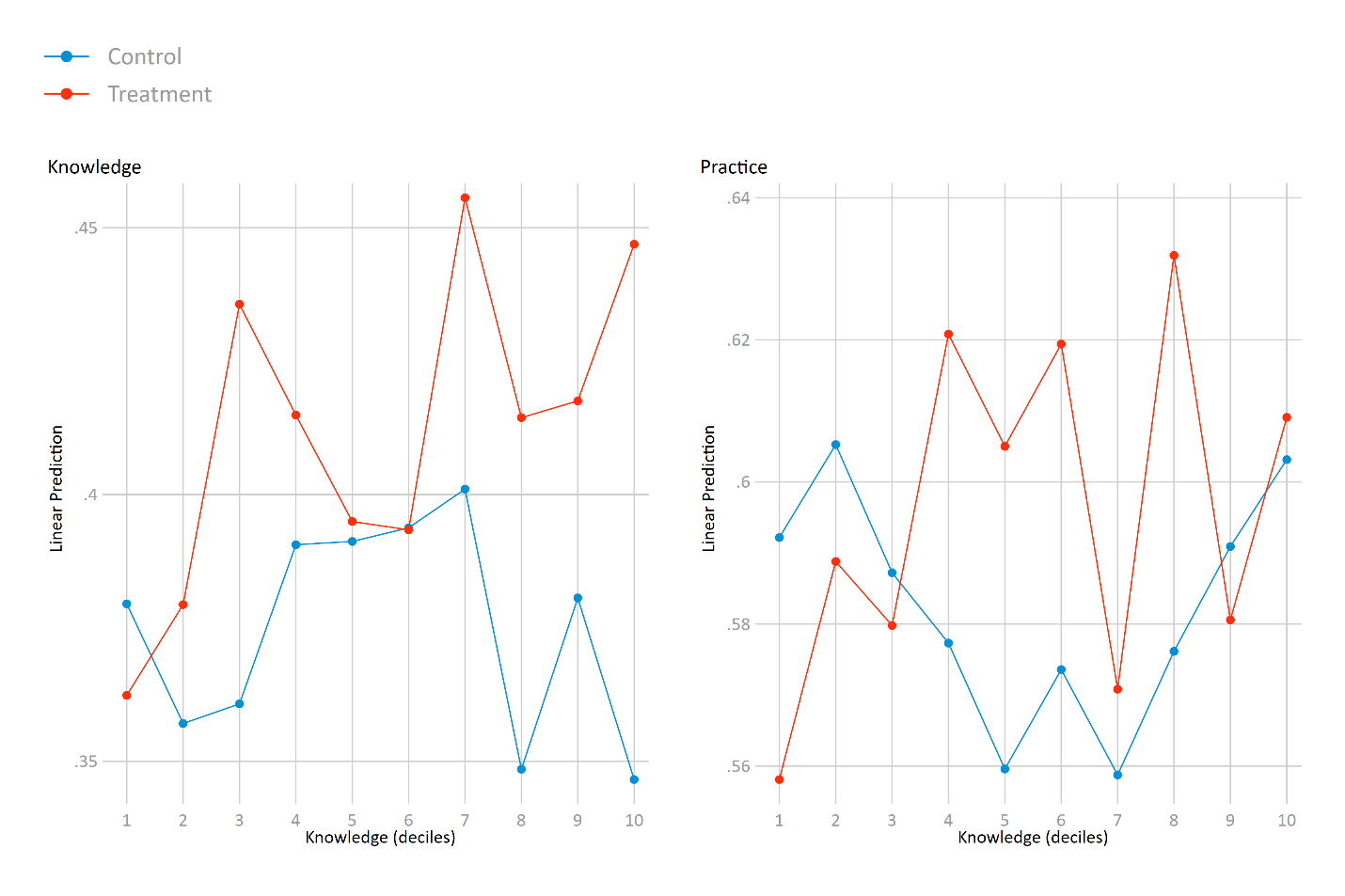


**S7 Figure. Short-term Effects by Education**


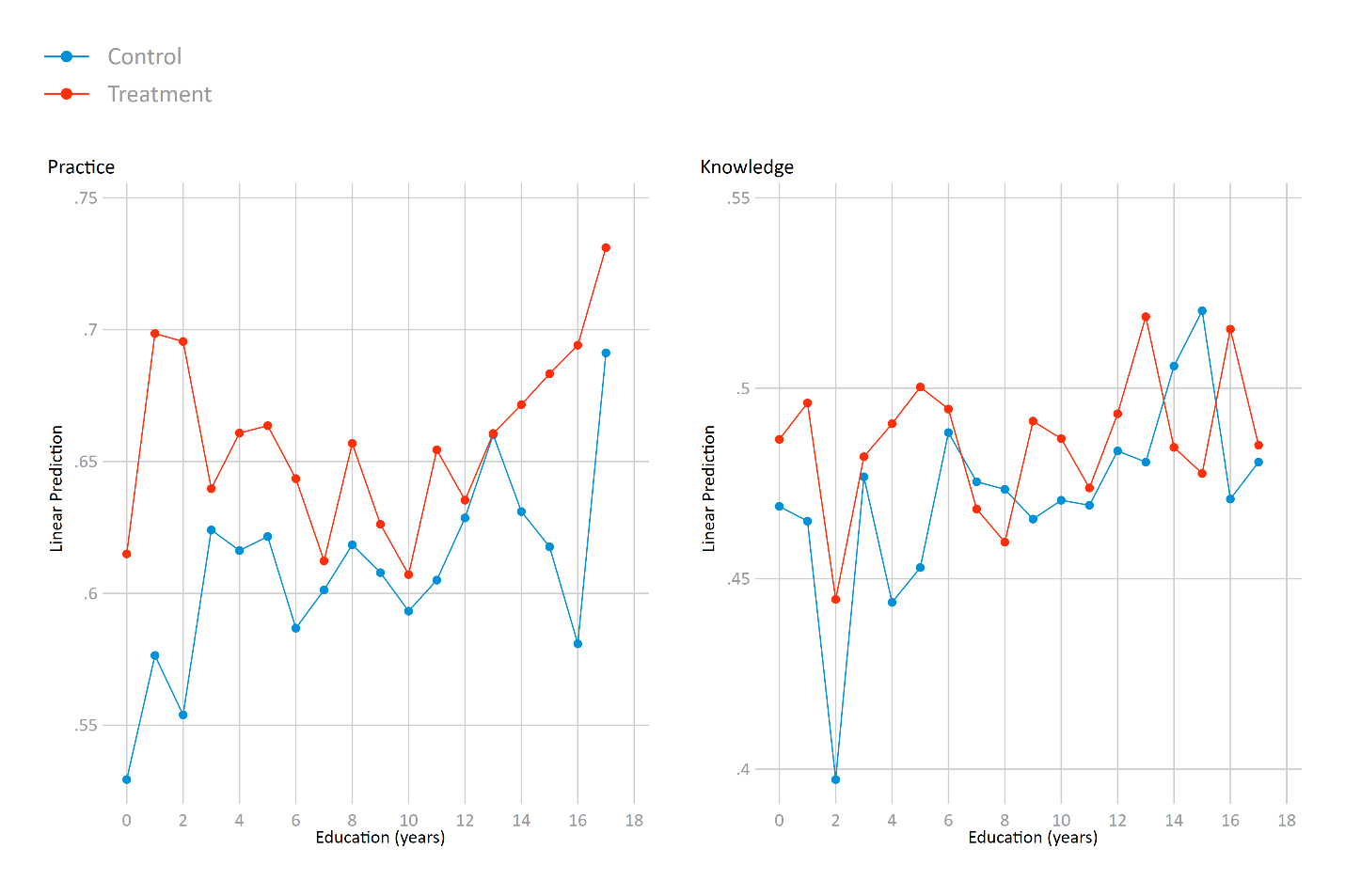


**S8 Figure. Long-term Effects by Education**

**
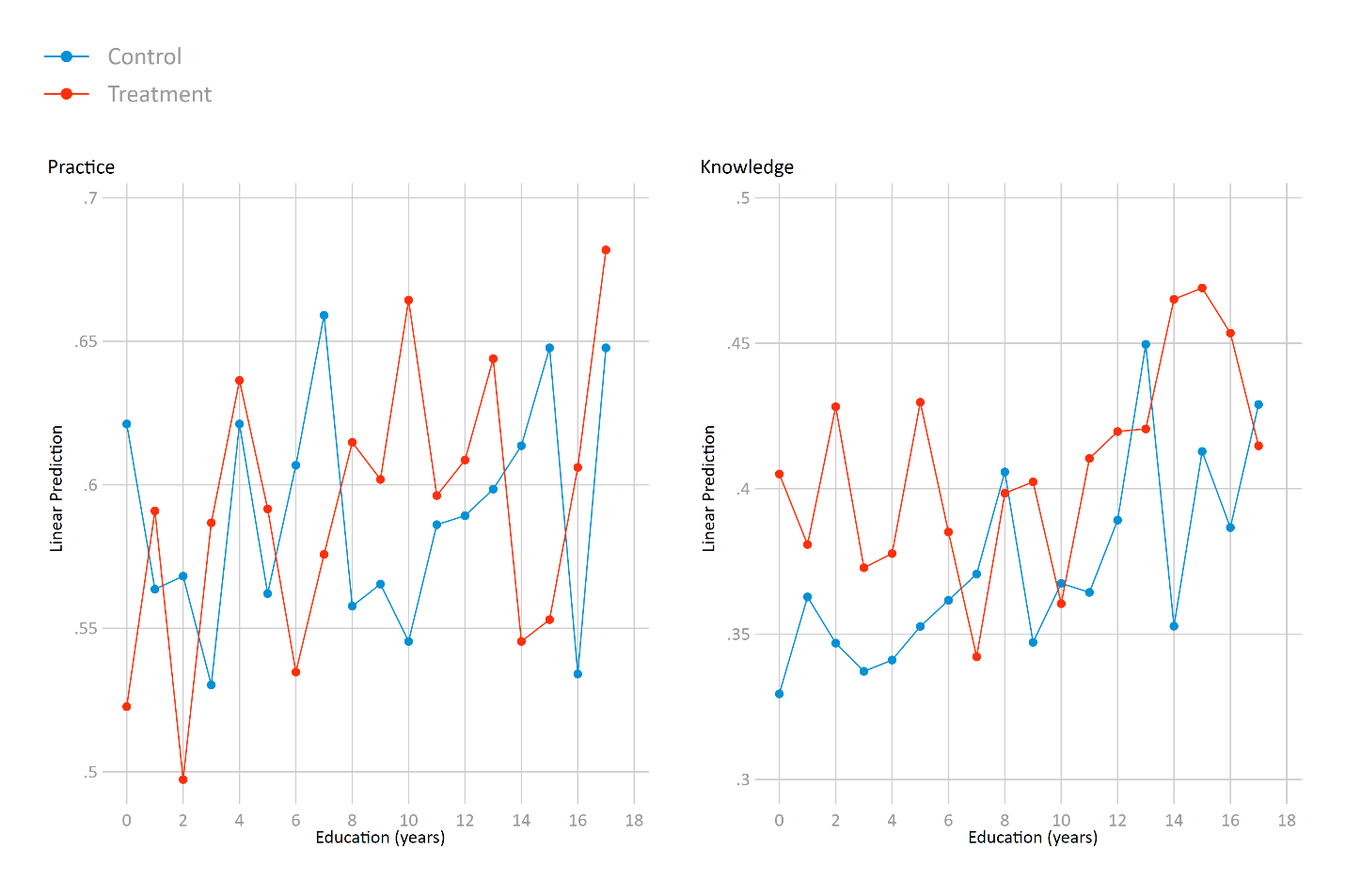
**
